# Evaluating Behavioral Management Practices for Laboratory Nonhuman Primates: An International Survey

**DOI:** 10.3390/ani16010138

**Published:** 2026-01-03

**Authors:** Kate C. Baker, Fiona R. Sewell, Mark J. Prescott

**Affiliations:** 1 Tulane National Biomedical Research Center, 18703 Three Rivers Road, Covington, LA 70433, USA; 2 National Centre for the Replacement, Refinement and Reduction of Animals in Research (NC3Rs), London NW1 2BE, UK

**Keywords:** animal welfare, behavioral management, enclosures, environmental enrichment, floor substrates, macaques, nursery rearing, psychological well-being, social housing, refinement, 3Rs

## Abstract

The largest survey to date of behavioral management practices among facilities breeding and housing nonhuman primates (NHPs) for research was conducted from 2019 to 2020. Its primary objective was to analyze and compare practices across the European Union (EU), the United Kingdom (UK), and the United States of America (US), identify regional differences, and highlight opportunities for refinement. Detailed information was captured on the management of NHPs at 49 facilities in these regions. Most respondents reported the number of NHPs at their institutions, which totaled 50,842. The findings suggest large variation in NHP behavioral management practices between world regions. Facilities in the UK and EU are at the forefront of best practice in many components of behavioral management. These involve the proportion of NHP populations housed socially, the age at which infants are removed from dams, the provision of pen enclosures, caging exceeding the regulatory/accreditation minimum floor space, and destructible enrichment (including floor substrates). Comparisons with the results of previous surveys conducted in the US in 2003 and 2014 show positive changes and progress over time, including a greater proportion of facilities providing access to exercise enclosures with more frequent implementation. Some previously reported impediments to social housing involving clinical and staffing constraints as well as scientific instrumentation eased over time. While there were increases in the use of social housing for some species, there was no increase across all species over the six-year interval since 2014. The proportion of US facilities providing destructible enrichment for caged NHPs remained the same. Importantly, overall, the data demonstrate that, with knowledge transfer and sufficient resources, perceived constraints on key elements of behavioral management can be overcome to improve NHP welfare and facilitate good science. The findings have been used to identify and discuss recommendations for best practice to improve animal welfare.

## Simple Summary

Nonhuman primates are used in biomedical research worldwide, and improving their welfare is essential for ethical and scientific reasons. Behavioral management practices, such as social housing, environmental enrichment, and appropriate enclosure design, help animals express natural behaviors and reduce stress, but approaches vary internationally. This study reports the largest survey of behavioral management for research laboratory primates, gathering information from 49 facilities in the EU, UK and US. The survey examined key areas of social housing, nursery rearing, enclosure size and design, and types of enrichment. For the US, comparisons were made with previous surveys to identify changes over time, providing a unique perspective on long-term trends. Results show notable regional differences. EU and UK facilities generally reported higher standards, including more social housing and larger enclosures, while US facilities demonstrated progress in some areas, such as exercise enclosures, but with opportunities for further improvement in others. Cost, space, and staffing were commonly identified as challenges in the US. These findings provide valuable insights into recent practices and highlight opportunities for improvement through knowledge sharing and harmonized approaches. Strengthening behavioral management benefits animal welfare and enhances the quality of research, supporting global efforts to refine care and promote best practice.

## 1. Introduction

Behavioral management is a holistic set of methods for improving animal care and welfare. Key components of modern nonhuman primate (NHP) behavioral management programs include full contact social housing, a comprehensive dynamic and effective evidence-based enrichment plan, animal training methods based on voluntary cooperation and positive reinforcement, facility design and husbandry practices that support welfare, and attention to the effectiveness of programs and outcomes for individual animals [1,2,3,4]. Standards and practices for the behavioral management of NHPs used and bred for research have evolved over time, guided by factors such as the increasingly large body of research literature on this topic and increased awareness of the role of good welfare practices in producing high quality science [5,6,7,8]. Good behavioral management can reduce the confounding effects of stress on experimental endpoints and improve scientific outcomes by reducing inter-individual variation and enhancing model validity [9,10].

While behavioral management practices have evolved over time, most evidence comes from single-region studies. For example, a 2003 survey of 22 NHP facilities in the United States (US) and a follow up survey in 2014 found that over 90% had modified their programs over the prior several years, often driven by external site visits they had received, regulatory standards, and increased access to information. These surveys demonstrated progress, particularly in social housing and highlighted the role of knowledge sharing in advancing animal welfare, in line with the 3Rs (replacement, reduction and refinement of animals in research) [1,11,12,13,14]. However, comparable international data are lacking, leaving a gap in understanding how practices differ across regions and where opportunities for global harmonization exist. To address this, an international survey was conducted in 2019–2020 among facilities breeding and housing NHPs for research, as part of a transatlantic collaboration between the Tulane National Biomedical Research Center and United Kingdom (UK) National Centre for the Replacement, Refinement and Reduction of Animals in Research (NC3Rs). The survey was designed not only to document current practices but also to evaluate attitudes, constraints, and opportunities for refinement in behavioral management across regions.

The survey included 77 questions covering a broad range of behavioral management practices. This manuscript focuses on six key domains most relevant to welfare and refinement: social housing, nursery rearing, enclosure size and design (including exercise enclosures), destructible enrichment, sensory enrichment, and cognitive enrichment. These areas represent core components of behavioral management programs and offer the greatest opportunities for refinement and harmonization across regions. Results are presented with comparisons across regions and over time, alongside an analysis of constraints and opportunities for refinement.

To interpret these findings, it is important to consider the regulatory context shaping behavioral management practices globally. Most countries have regulations and policies pertaining to the use of animals in research and whilst these are often well aligned across regions in terms of performance requirements such as social housing of NHPs, they differ considerably in their engineering specifications and mechanisms of enforcement and oversight. There has been increasing attention paid to harmonizing the care and use of laboratory animals, primarily through accreditation, most commonly via the Association for Assessment and Accreditation of Laboratory Animal Care International (AAALAC). The AAALAC Council and assessors use the *Guide for the Care and Use of Laboratory Animals* (Institute of Laboratory Animal Research, ILAR Guide) [15] as the primary standard for evaluation of NHP care and use programs in the US, Pacific Rim, and rest of the world. However, the EU Directive 2010/63/EU [16] is used by AAALAC in those member countries where it applies. Also of note in the EU is Appendix A to the Council of Europe Convention ETS 123, revised in 2006, which, whilst not legally binding, provides guidance on the accommodation and care of laboratory animals beyond the minimum enclosure sizes and space allocations specified in the EU Directive [17]. Additionally, in the UK and some other countries, research funding organizations have defined their own standards of NHP accommodation, care and use, and have made their implementation a condition of grant funding [18].

Given these regulatory differences, awareness of the variability of behavioral management practices can help stakeholders in evaluating and benchmarking their own programs, provide an expanded view of what is feasible when caring for NHPs in the laboratory environment and identify opportunities for harmonization. Against this regulatory backdrop, this present survey queried a large number of elements of behavioral management. Here, we present findings for the selected domains, examining regional differences in implementation and their relationship to regulatory frameworks in the EU, UK, and US. These findings are used to highlight opportunities for refinement and inform recommendations for best practice. While not intended as a comprehensive scientific review, relevant scientific evidence and literature are referred to where available, with a focus on macaques and inclusion of other species where possible.

To evaluate potential changes over time, we compare our findings to two prior surveys of the behavioral management practices for laboratory NHP species in the US, implemented in 2003 [19] and 2014 [5]. These three surveys present a unique opportunity to examine long-term trends in behavioral management in the US and components such as social housing, exercise enclosures, destructible enrichment (including floor substrates), and sensory enrichment and factors constraining implementation. Other components are not addressed because they were not queried in earlier surveys.

The primary objective of this study was to analyze and compare behavioral management practices for laboratory-housed NHPs across the EU, UK and US.

Secondary objectives were to:

■ Identify regional differences in practices and constraints to implementation;
■ Compare US practices over time using data from previous surveys;
■ Use findings to inform recommendations for best practice and refinement.

## 2. Materials and Methods

### 2.1. International Behavioral Management Survey 2020

The survey comprised 77 structured questions (multiple choice and free text) and was administered online through SurveyMonkey (SurveyMonkey Inc., San Mateo, CA, USA, version current in 2019; see Supplementary Information). It was developed by the authors (KCB and MJP), adapted from previous surveys [5,19] and piloted by colleagues. A downloadable version was provided to allow participants to prepare answers offline. Each facility submitted a single consolidated response from personnel directly involved in the care, use, or breeding of NHPs, based on a recent point in time. Responses from institutions of the same type and region were checked to avoid duplication.

Between November 2019 and February 2020, invitations were sent to over 80 targeted employees at NHP research facilities internationally. Recipients were identified through the NC3Rs NHP welfare network, the UK Network for Named Animal Care and Welfare Officers (NACWOs), and the Behavioral Management Consortium of the US National Primate Research Centers (NPRCs). Open calls were also shared via the NC3Rs website, newsletter and social media channels, the 2019 NC3Rs Primate Welfare Meeting, the NC3Rs Macaque website (https://macaques.nc3rs.org.uk, accessed on 20 December 2025), LinkedIn groups (Association of Primate Veterinarians, European Primate Veterinarians, NHP Toxicology); and primatological societies (American Society of Primatologists, International Primatological Society).

The survey closed on 21 February 2020 yielding 49 responses, which were downloaded to Microsoft Excel (Microsoft Corporation, Redmond, WA, USA; Office 365 version current in early 2020; RRID:SCR_016137) for analysis. Free-text answers that could potentially identify facilities were redacted. Since some respondents did not answer all questions, the number of responses per question varied and is indicated where relevant.

The survey was conducted anonymously, with no directly identifiable personal or institutional data collected. Participants provided informed consent prior to submission. Formal ethics committee review was not sought at the time because the study involved anonymous responses only and no new animal procedures, although we acknowledge that current best practice would be to seek review for similar studies. A data management plan outlining how research data would be managed, stored, shared and protected, was available on request.

The survey used a convenience-based sampling method, which has limitations. Results may not fully represent the broader NHP research community due to non-random sampling, unknown response rate, and potential response bias. While response bias is a limitation of all survey-based research, there is no practical way to obtain this type of data at scale. Responses were analyzed using descriptive statistics (percentages, medians, ranges) to summarize trends across facilities and regions; inferential tests were not applied due to sample size and survey design.

For interpretation, findings were considered in the context of regional regulations, including EU Directive 2010/63/EU [16], the UK Code of Practice for the Housing and Care of Animals Bred, Supplied or Used for Scientific Purposes [20], and the US Department of Agriculture (USDA) Animal Welfare Act [21]. The UK Code of Practice includes both legally binding requirements and advisory guidance. Where UK regulations are referenced, the focus is on the legal mandates to ensure consistency with EU and US frameworks; advisory guidance is noted where applicable.

### 2.2. Prior Surveys; Tracking of Changes in US Facilities over Time

US data from the 2020 survey, along with two previous surveys on the behavioral management of laboratory NHPs conducted in 2003 [19] and 2014 [5], were used to examine long-term changes in the US. Elements surveyed included social housing, exercise enclosures, destructible enrichment (including floor substrates), sensory enrichment, and factors constraining implementation. However, not all components of behavioral management were queried at all three time points or at the same level of detail, so it was not possible to track changes in every element surveyed in 2020. Where comparisons were feasible, these are included in the relevant sections below. For all surveys, respondents were assured anonymity and informed that data would be used only for professional presentation or publication. Since responses in the most recent survey were anonymous, changes reflect overall trends rather than changes within individual institutions.

#### 2.2.1. Survey of US Facilities 2003 [19]

This survey included 56 questions. In June 2003, questionnaires were sent to individuals who either worked directly with NHPs in research facilities or performed management oversight of enrichment programs. Questionnaires were returned by 22 institutions.

#### 2.2.2. Survey of US Facilities 2014 [5]

This survey included 79 questions and was sent via email to heads of behavioral management programs when known to the authors or otherwise directed to senior management with a request to forward the questionnaire to the appropriate individuals. Additional participants were identified through professional networks, membership directories (American Society of Primatology and Association of Primate Veterinarians), LinkedIn and a notice posted to the Laboratory Animal Refinement Forum (awionline.org). Questionnaires were returned by 41 facilities.

## 3. Results

The results of the 2020 survey were obtained from facilities in Belgium (1), France (7), Germany (2), Hungary (1), Sweden (1), Switzerland (1), the UK (7), and the US (29). Responses were also received from Canada (2), India (1), Israel (1), and St Kitts (2), but these regions differ substantially in regulatory and cultural standards and had very small sample sizes, so they were excluded from the dataset. For comparing international regions, the remaining countries were collapsed into three categories: EU (13), UK (7), and US (29). For ease, and since similar regulations are followed, Switzerland was grouped with the EU despite its status as a bilateral partner rather than a member.

Forty-four of the 49 facilities provided information on the animals comprising their NHP population (12 EU, 6 UK, and 26 US). Summed across the institutions providing animal numbers, facilities in the EU reported 2799, the UK 676, and the US 47,367, for a total of 50,842 NHPs. However, since not all facilities reported the number of animals they housed, the total number of animals represented in the survey will be larger and cannot be determined. NHP populations ranged from 2 to 7571 with an overall median of 264. Regional medians were 89 in the EU (range 11–999), 28 in the UK (range 10–500), and 1377 in the US (range 2–7571).

Respondents report the housing of the following taxa: rhesus macaques (30,332), long-tailed/cynomolgus macaques (12,817), African green monkeys (1656), baboons (1609), pig-tailed macaques (1023), common marmosets (1404), owl monkeys (509), common squirrel monkeys (479), Japanese macaques (349), mangabeys (173), capuchins (135), and titi monkeys (102) (Table 1). Five respondents reported numbers without attribution to a species, totaling 254 NHPs.

**Key results at a glance:**

Findings reveal substantial regional differences and highlight opportunities for refinement across six core domains, with US trends tracked over time where possible:

■ **Social housing:** Overall, 81% of NHPs were housed socially, with higher levels in the EU (93%) and UK (96%) than in the US (80%). For indoor-housed animals only, social housing was 89% (EU), 96% (UK), and 65% (US).
■ **Nursery rearing:** Rare in the EU but common in the US; minimum weaning ages ranged from birth to nine months, with 5 US facilities reporting separation at birth and 2 before six months.
■ **Enclosures:** 46% of facilities provided more than the required minimum floor space to all NHPs (EU and UK: 83%; US: 21%). EU-style pens were used by 56% of facilities overall, including all UK facilities and 37% of US facilities. Exercise enclosures were used by 55% of facilities for caged NHPs, most commonly in the US (67%).
■ **Destructible enrichment:** For caged NHPs, substrates (e.g., bedding or floor material for foraging) were used by 61% of facilities (EU 90%, UK 100%, US 38%), and other destructibles (e.g., paper, cardboard) were provided by 90% of facilities (EU and UK: 100%; US: 85%).
■ **Sensory enrichment:** Techniques such as music, video, or aromas were used by 90% of facilities for caged NHPs (EU: 90%, UK: 80%, US: 92%) and 52% for other enclosure types (EU: 80%, UK: 33%, US: 47%); cost and staffing were the main constraints.
■ **Cognitive enrichment:** Techniques that provide challenging tasks to engage intelligence (e.g., puzzle feeders, computer-based tasks) were implemented by 91% of facilities for caged NHPs (EU: 91%, UK: 83%, US: 92%), with provision to all animals at 46% of those facilities. For other enclosure types, cognitive enrichment was used by 57% of facilities (EU: 80%, UK: 100%, US: 47%). Time and cost were the most cited barriers and scientific exemptions were rare.
■ **US trends over time:** Since 2014, exercise enclosure use increased (from 50% to 67%), but overall social housing levels remained unchanged (65%), despite improvements for some species.

### 3.1. Social Housing

Facilities were queried regarding their use of various social settings, management techniques associated with social housing, and factors constraining the use of social housing—both constraints that fall under regulatory and accreditation standards and practical issues that are not addressed under these standards. For context, extracts from regional regulations relevant to the use of social housing are provided in Table 2.

**Key findings on social housing are summarized below:**

■ **Overall prevalence:** Social housing was widely implemented but varied by region, with EU and UK facilities consistently reporting higher levels than US facilities. Indoor-housed animals showed the greatest disparity.
■ **Techniques used to introduce social housing:** Most facilities (>75%) reported using compatibility data from suppliers and employing visual access or protected contact during introductions. EU facilities showed slightly greater reliance on protected contact than visual access, while UK facilities used these techniques less often despite high overall social housing levels. Observation of affiliative behavior before full contact was also reported, though slightly less common.
■ **Constraints to social housing:** Lack of social partners was the most cited constraint across all regions. Scientific exemptions were reported by a larger proportion of US facilities (83%) than EU (60%) or UK (33%). Practical issues such as space and staffing were also more common in the US.
■ **US trends over time:** No overall increase in social housing since 2014 (65%), despite improvements for some species. Techniques such as protected contact decreased slightly, while use of neutral cages increased.

Further details, including species-level data and regulatory context, follow below.

The following distinctions were made regarding social housing:

Restricted social housing:

■ Single housing: NHPs have visual and olfactory contact with like or compatible species, but without physical contact. Note, in the absence of visual and olfactory contact, the housing would be termed ‘isolation housing’, which was not queried in this survey.
■ Protected contact: individuals are separated via a barrier consisting of bars or mesh that permit social contact but not entry into another individual’s enclosure.
■ Intermittent pair housing: individuals are kept in a shared space with a partner but on a periodic basis (e.g., part of a day or week).

Full social housing:

■ Continuous pair housing: individuals kept in a shared space with one partner.
■ Indoor group housing.
■ Indoor group housing with access to the outdoors.
■ Outdoor group housing.

For the purposes of calculating the use of social housing in this survey, protected and intermittent contact were considered a form of single housing. Characterizing them in this way is based upon language in the ILAR Guide [15] which encourages the provision of tactile contact to singly housed individuals- and characterizes intermittent contact as “social experience” rather than social housing per se.

#### 3.1.1. Proportion of NHPs Housed Socially

Most facilities employed more than one type of housing. Over all respondents, 77% of facilities housed animals in more than one enclosure type, with EU values at 85%, UK 71% and US 75%. The proportion of animals of each species housed socially across all housing types in the different regions is reported in Table 3. Further details on the proportion of NHPs of each species housed in each of the seven housing categories are presented in Table S1.

Across all facilities providing information permitting a quantification of social housing, 81% of NHPs were housed socially. A greater proportion of NHPs in the EU (93%) and UK (96%) were housed socially than in the US (80%) (see Table 3 for a breakdown by species). These values represent NHPs housed in both outdoor and indoor enclosures in the EU and US, but in the UK these include only indoor housed NHPs, since none of the UK institutions reported housing NHPs outdoors.

For indoor-housed NHPs only (i.e., excluding animals housed in indoor group housing with access to the outdoors or outdoor group housing), the regional contrast in levels of social housing were considerably greater (EU: 89%, UK: 96%, US: 65%); (Table S2). In the US, the fact that social housing was less prevalent among indoor-housed animals than across animals in all housing types may relate to the fact that in the US NHPs housed outdoors are often part of breeding colonies, which necessitate social housing, whereas NHP populations housed indoors tend to be used in biomedical research, where single housing is more likely to occur.

The above values aggregate caging and indoor group housing, even though caging often includes single housing, whereas indoor group housing is specifically designed for social housing. When examining caged NHPs only, levels of social housing were indeed lower overall. The regional differences seen with other comparisons (i.e., all NHPs and NHPs housed inside) were still apparent when quantifying social housing among only caged NHPs (EU: 76%, UK: 69%, US: 58%) (Table S3) although the disparity was narrower than the values across all housing types or indoor housing.

Examining regional differences in the housing of particular species was hampered by the fact that many species housed in cages were represented only in the US. Interestingly, the proportion of social housing for rhesus macaques housed in cages was similar across the three regions (47–49%). This species comprised the largest number of NHPs in this dataset, in terms of both the number of institutions reporting on their housing and the number of animals whose housing was reported. Among other species, there were higher levels of social housing in cages in the EU than in the US, most dramatically among baboons (EU: 86%, US: 39%) (Table S3). However, it should be noted that the EU figure represented only 21 animals from one institution. Some of the differences between regions may pertain to species housed only in the US (Table 3).

#### 3.1.2. Techniques Associated with Social Housing

Forty-eight facilities responded regarding the techniques used when introducing NHPs for social housing (13 EU, 7 UK, and 28 US). Thirteen techniques were queried and reported in Table 4. Most commonly cited techniques included the use of compatibility or hierarchy data from suppliers, visual or protected contact access before full physical interaction, and the requirement that affiliation be observed in order to deem introductions successful. Rarest were contraception, manipulation of enclosure location or size, canine blunting, and medications.

There was considerable variability across regions. The majority of techniques queried were reported by a smaller proportion of UK facilities than other regions, but since the UK showed the highest levels of social housing, there is no indication that the absence of these methods reduces the overall success of social housing programs. The relative rarity of techniques relating to enclosure size or familiarity to potential group members may relate in part to the larger routine size and complexity of enclosures in which the UK NHP population resides.

Many of the techniques queried in this survey have been widely advocated, and there is research available in the literature that may guide program decisions and speak to downstream effects of changing practices, such as the exacerbation or alleviation of challenges to the implementation of social housing. We discuss several of these techniques below. However, there are knowledge gaps and a clear need for future research. An awareness of alternatives, ongoing research, and a recognition that common practice is not always based on adequate scientific evaluation could encourage institutions to be more flexible in their approaches to introductions with the aim of maximizing social management outcomes.

Broadly, most of the published research on social housing has been performed on macaques, which is not surprising given their predominance in laboratories, the published literature [22] and the results of this survey (see Table 1). It is important, of course, not to generalize findings to taxa with different social organization in the wild. In addition, some of the research cited below tests techniques in the context of pair housing but not larger groups.

The use of compatibility or hierarchy data from suppliers was one of the most widespread techniques reported; by over three quarters of facilities, lowest in the UK and highest in the US (Table 4). When animals are transported with individuals known to be familiar and compatible, such data can significantly enhance the success of social housing and introductions. The lower use of supplier-provided compatibility data in the UK (57%) compared to the US (86%) likely reflects the fact that UK facilities are typically supplied with pre-established compatible pairs or groups, reducing the need to request or rely on additional data. Moreover, access to compatibility or hierarchy information enables animals to be housed in a social setting as swiftly as possible after arrival at a new facility. This practice is particularly important given that transport-related stress—well documented across a wide variety of taxa—can lead to significant and persistent physiological changes. In some species, age groups, or transport durations, this stress may be mitigated by prompt social housing [23,24,25,26,27,28,29,30,31].

In contrast to simply placing NHPs into pairs or groups, gradual-step introductions include placement in full contact only after implementing phases involving visual access (through plexiglass or fine mesh) and/or protected contact. Using visual access as a preliminary step in a social introduction was reported by about three quarters of facilities, more commonly in the US and EU than the UK (Table 4). Across all regions, protected contact was employed as an intermediate step at the same level as visual access. EU facilities reported implementing protected contact more commonly than visual access.

Aggregating visual and protected contact, 85% of facilities use at least one technique (EU: 92%, UK: 57%, US: 89%). Eighty percent of facilities that use one intermediate technique use both. In keeping with this pattern, publications indicate that some institutions employ both steps during rhesus macaque pair formation [32,33], whilst some do not [34,35]. Comparisons have been conducted in the context of adding adult male rhesus macaques to breeding groups. Providing visual and physically limited contact is associated with higher levels of success [36,37,38].

The gradual steps method is widely advocated and considered beneficial, particularly with respect to caged NHPs. However, the potential benefits of a gradual-step process must be balanced against possible welfare risks relating to prolonged frustration leading to aggression, or the expression of non-contact aggression without risks of escalation. Gradual-step instructions can require considerable personnel time, which was reported by some facilities as a constraint to implementing social housing, particularly in the US (Table 3). There have been few direct comparisons of outcomes comparing the use of gradual steps to other methods [39]. Also, the success of gradual-step introduction strategies is likely to vary between species. For example, comparisons of pairing methods for vervet monkeys failed to detect benefits of gradual introductions, and in fact revealed relatively poor outcomes at some facilities using this technique [40]. In addition, findings concerning long-term pair housing in rhesus and long-tailed macaques found that rhesus macaques benefit less in protected contact than in full contact, whereas long-tailed macaques did not show this difference [41]. This finding suggests that the use of protected contact during introductions may influence outcomes differently in the two species. There are likely to be considerable species, sex, and age differences as to the necessity for or success of using gradual steps, but they have yet to be explored. Also needed are scientific evaluations of varying durations of restricted access before allowing full contact.

Requiring the observation of affiliation to conclude that an introduction has been successful was reported by approximately two-thirds of facilities, used almost universally in the EU, and a little over half in the UK and US (Table 4). Among social housing techniques included in the survey, this technique showed the greatest disparity across regions. This is one of the most important techniques to scrutinize as it can bear on the most commonly cited constraint to the use of social housing, the absence of social partners. There are several studies that bear on this practice. Grooming, a hallmark measure of affiliation, has been found to be a relatively poor predictor of the outcome of rhesus macaque pair introduction and longer-term compatibility [33,42]. Furthermore, in rhesus macaques, grooming has been shown to occur at low levels during the monitoring of pair introductions [33,42,43], which, of course, occur over brief periods that are not sufficient for supporting the idea that a behavior is absent.

Employing inaccurate behavioral predictors of pairing outcomes may result in the termination of pairing attempts that would have been successful, which, in the absence of alternative partners, would leave a NHP singly housed unnecessarily. This occurrence can be minimized by ensuring that personnel are aware that tolerance of proximity, as well as joint threatening of others, which occur at higher levels than grooming, are more useful and accurate outcome predictors [33]. Again, care must be taken to avoid generalizing findings from one species to other taxa. Nonetheless, taking a fresh look at decision-making and testing of alternative paradigms may lead to more productive introduction attempts and avoid unnecessary single housing. If there are alternative social partners, terminating an introduction will lead to additional introduction attempts, all bearing some risk. Each introduction involves some level of stress for the animal and risks a poor outcome. While they may be successful, re-attempts or introduction attempts with alternative or multiple partners can have potential negative impacts on welfare, suggesting that separating partners using inaccurate behavioral predictors imposes a cost regardless of the availability of partners.

The use of neutral space was queried for caged introductions and group formations occurring in other enclosure types. Introductions were reported to occur in neutral cages by approximately one half of facilities, least commonly in the UK (Table 4). For introductions involving enclosures other than cages, it was reported more rarely, and again, least commonly in the UK. With regard to conducting introductions in enclosures that vary in size from primary enclosures, the survey did not distinguish between cages and other types of enclosures. Less than a quarter of facilities reported conducting introductions in enclosures larger than primary housing, less common in the UK than the EU and US. Using smaller enclosures was reported more rarely, at only one in ten facilities, but in this case, most commonly in the UK.

There is surprisingly little evidence regarding outcomes of any of these techniques. With regard to introductions, to the authors’ knowledge, the effect of expanded enclosure size on the outcome of social introduction was reported only once, finding improved outcomes of African green monkey pairing [44]. We can also extrapolate from studies of individuals in stable social situations to infer benefits of expanded space for introductions [45]. However, to the authors’ knowledge, these techniques are more grounded in knowledge of NHP behavior rather than research. For example, it is reasonable to suggest that a neutral enclosure would prevent territorial aggression that would negatively affect introduction outcomes in species that are territorial in the wild. Similarly, it is reasonable to suggest that increased enclosure size would improve introduction outcomes due to providing for greater flight distance in recognition of the fact that no enclosure recapitulates the space used by NHPs in the wild. The authors are not aware of any studies of the use of smaller space for introductions, and it may be used relatively rarely due to the same considerations underlying the use of expanded space. Smaller enclosures may, however, facilitate rapid separation of animals that are fighting. Of course, increased space is novel space, so these techniques are intertwined. It is likely that facilities are constrained from employing novel or differently sized enclosures for some of the same constraints to the use of social housing, such as housing or space limitations.

Contraceptive techniques can include medication (delivered via oral medication, injections, or implants), vasectomies, removal of gonads [46,47], and formation of same-sex social groups. The use of contraception was reported as a part of social management at 29% of facilities, in approximately a third of facilities in the EU and US, and at much lower levels in the UK. Studies in several species have found that some forms of contraception can have positive effects on individual behavior and group dynamics [46]. Importantly, contraception can be used to expand the pool of potential companions for singly housed animals (i.e., when there are no same-sex companions available).

Both medical contraceptive techniques and formation of same sex groups have advantages and disadvantages. Using medical contraception is superior because it can permit compatible groups to remain stable when breeding is not desired, rather than requiring that individuals experience group disbanding or placement in smaller and less socially enriching housing settings. In addition, adult males housed in what would otherwise be all-female groups may serve a beneficial social role in certain species; for example, as conflict managers, thereby increasing group stability and reducing trauma (wounding) rates [48]. On the other hand, same sex groups avoid the need to subject animals to surgical procedures for medical contraception and the potential research confounds that may be associated with these. However, more enclosures are required for housing of same sex groups which may limit their implementation in some facilities.

Though rarely reported in this survey, canine blunting is a technique aiming to reduce injurious aggression. However, this approach is not supported by the scientific literature and is actively discouraged by regulatory and professional bodies. In fact, in rhesus macaques, it has been shown only to alter the nature of wounds rather than their severity [49,50] and can have adverse effects on dentition [49,51]. In the US, the USDA Animal and Plant Health Inspection Service, the American Veterinary Medical Association, Association of Primate Veterinarians, as well as the International Primatological Society, do not endorse reduction or extraction of canine teeth in NHPs unless required for medical treatment or approved scientific research [21,52,53].

Using medication as part of social management was reported very rarely, and not at all in the UK. The survey tool did not ask respondents for information regarding the type of medication used. However, employing medication as a tool for supporting social housing has been studied quite recently, with respect to guanfacine [54] and diazepam [39,55]. Outside of an introduction setting, endogenous oxytocin is associated with prosocial behavior in a number of NHP species (e.g., rhesus macaques [56], cotton-top tamarins [57], tufted capuchins [58], marmosets [59], titi monkeys [60], and hamadryas and Anubis baboons [61]. Medication can also be employed to reduce agonism (rhesus macaques [62]). To the authors’ knowledge, there are no scientific papers evaluating the use of exogenous oxytocin in social introductions.

#### 3.1.3. Factors Constraining the Use of Social Housing

Forty-four facilities provided information on the reasons preventing NHPs from being housed socially (12 EU, 6 UK, and 26 US). Response options included reasons outlined in regulations. As outlined in Table 2, regulations in all three regions provide exemptions from social housing, based upon reasons relating to scientific restrictions, behavioral/animal-welfare or animal-health (clinical). However, since single housing has been documented to occur for reasons that fall outside of the scope of these exemptions [5,19], non-scientific reasons (i.e., practical constraints) were also captured in the survey. All facilities reported that social housing was restricted by all four of these categories (Table 5). Clinical and behavioral constraints were reported at a lower level in the EU than the other regions, whereas the US reported scientific constraints more often than the EU and UK. Practical and scientific constraints were examined in more detail, with the results and potential solutions to these constraints discussed below.

Across all regions, the most cited practical challenge, and indeed the biggest challenge overall, was the unavailability of social partners. No other issue approached this prevalence. Potential strategies for increasing the pool of potential partners are discussed below in relation to scientific constraints. No UK facility cited constraints associated with time/staffing, housing, space, or delays associated with obtaining necessary information from investigators. No EU facilities cited costs as a constraint. However, US facilities reported costs, housing and time/staffing constraints at a high level. With the exception of concerns over negative consequences, which the EU reported at a higher level, all US facilities reported all constraints at a higher prevalence than the other regions. Encouragingly, no facility indicated a belief of a lack of benefit as a rationale for single housing, suggesting that social housing is accepted as a best practice.

In addition to querying the level of overall scientific exemptions, the survey queried the relationship between social housing and several specific scientific justifications (Table 5). The survey tool required respondents to indicate whether certain procedures or areas of research were performed at their institutions. Each value below is calculated only from facilities for whom the procedure or specific justification was relevant. For example, the use of social housing for tethered animals was quantified only from data from facilities that tethered research subjects. Among all the specific scientific exemptions queried, UK facilities only reported one—using single housing for urine collection. When comparing the EU and US, the majority of scientific constraints were reported by both regions. Amongst the most prevalent scientific reasons for single housing were concerns over cross-infection, urine collection and several forms of scientific instrumentation, most often tethering. The use of cranial implants was only cited as a constraint in the US, and at very low levels, and jacket use was only cited in the EU.

Urine collection was reported as a constraint in all regions and was the only scientific constraint reported by UK institutions. Various refinements are available to minimize the duration and impact of single housing for the purposes of urine collection, such as employing brief separations from group members using protected contact [63] as a substitute for individual metabolism cages. In fact, single housing for urinalysis purposes may not always be required. Positive reinforcement training [64,65] can be used with animals in group housing. Furthermore, group data (i.e., pooled urine from multiple animals) rather than individual data may be acceptable under some circumstances [12]. This may be the case particularly for ADME (Absorption, Distribution, Metabolism and Excretion) studies, where metabolism caging permitting group housing has been associated with radiation excretion profiles with the same means and variation as those calculated across singly housed individuals in traditional metabolic cages [66].

Cross-infection was cited as a constraint to social housing by half of EU facilities and three-quarters of the US facilities, but by none of the UK facilities. The risks from cross-infection vary widely depending on the pathogen and circumstance [6]. For example, horizontal transmission may occur more readily under some circumstances (e.g., Simian Immunodeficiency Virus (SIV) transmission through breast milk) than others, and with the risk also dependent on pathogen load and strain. In contrast, cross-transmission across socially housed groups has not been detected across a number of facilities which group-house animals on SIV, tuberculosis and other infectious disease experiments [6]. Research may benefit from balancing the risk of cross-infection against the potential consequences of single housing, including immune system perturbations, altered disease progression, increased variability in outcomes, and reduced translational relevance [67,68,69]. One step to maximize the use of social housing in the context of cross-infection is to limit its implementation to the shortest duration possible. It is unnecessary, for instance, to singly house NHPs for the entire duration of a study, even during the pre-infection period, when cross-infection is not a concern. Another is to synchronize study timelines across subjects when possible. Particularly where studies limit potential partners to those with the same viral status or to phases during which social housing is acceptable, infecting in an asynchronous fashion would reduce the pool of available social partners. Another step toward maximizing the use of social housing is to expedite the determination of the results of experimental infections, when these results will end the need for single housing. Batching of samples used to determine viral status can significantly lengthen the duration of single housing.

Measuring food or fluid consumption was reported as a constraint by more than half of US and EU facilities. Planning for pair housing and quantitative intake assessment methods should be considered early in the study design stage [6]. With appropriate care; even animals in studies utilizing food/fluid control protocols as motivators in behavioral or cognitive tasks can be housed with companions [70]. As with urine collection and tethering (see above); protected (or intermittent) contact may be another means for providing social contact opportunities. Where constraints relate to daytime research activities; social housing at night should be considered, during which individuals show high levels of huddling and embracing [71]. It is possible that post-operative hypothermia [72] can be attenuated due to this social contact. Microchip-automated feeders/waterers that release food or water to specific individuals are another, albeit expensive, option that can avoid the need for single housing.

Tethering was reported as a constraint by a third of EU facilities and almost two-thirds US facilities. However, caging systems permitting protected contact housing for tethered animals have been developed [73,74,75] which provide a social setting superior to single housing. But it is not clear how widely or successfully these systems are being used. The use of tethers may be avoided through vascular access ports for blood collection (possibly requiring positive reinforcement training), but this may be difficult to implement when continuous dosing or very frequent blood draws are required. The significantly lower rate at which institutions in the EU report tethers requiring scientific exemptions suggests that this is not a common obstacle to social housing. Housing tethered individuals with untethered individuals determined to be compatible prior to tethering would avoid the risks of tether entanglement and may be a strategy for providing tethered animals a social buffer that may facilitate acclimation [76] and minimize chronic stress [74]. This housing arrangement has been successfully implemented on a programmatic level [77].

Interestingly, neither collars nor eye coils were reported to constrain social housing in any region. The use of jackets was only reported as a constraint by a quarter of EU facilities (25%) and use of cranial implants only by a small proportion of US facilities (8%). Research suggests that single housing is not needed for jacketed individuals [8,78]. With respect to internal implants, responses may refer to a wide variety of implant types. A review of studies and a survey regarding the use of cardiac telemetry implants found the need for single housing to be unsupported in the literature and that the use of social housing was increasing for this type of implant [8,79].

#### 3.1.4. Changes in the Use of Social Housing in US Facilities

There have been improvements in the levels of social housing for indoor-housed NHPs in the US since 2003, rising from 46% to 64% in 2014, though found to be unchanged in 2020 (65%). This appears to be driven by the unchanging level of social housing in rhesus and long-tailed macaques, which made up 84% of the total number of NHPs housed indoors. In fact, levels of social housing for rhesus macaques was nearly identical at all three time points. While the use of social housing for long-tailed macaques increased significantly in the 11 years between 2003 and 2014, there was no further expansion during the subsequent six years. However, very large increases were seen between 2014 and 2020, for pig-tailed macaques, Japanese macaques, baboons, mangabeys, and squirrel monkeys, with decreases seen only for capuchins and marmosets (Table 6).

Whilst any improvements are encouraging, the lack of progress between 2014 and 2020 is disappointing, due to the benefits of social housing and the fact that it is firmly entrenched as a best practice and heavily emphasized by regulatory and accreditation bodies.

The surveys captured information regarding the constraints to implementation of social housing and therefore may provide insights regarding the apparent stagnation in reported use of social housing since 2014. First, the impact of scientific exemptions has grown. Also, unlike levels across all scientific exemptions, constraints pertaining to scientific instrumentation queried at both time points were cited less often in 2020 than 2014 (Table 7). In fact, no facility using jackets, eye coils, or collars reported that they constrained social housing. The use of tethers remained the top instrumentation constraint; however, its level was also lower in 2020 than in 2014. While changing practices with regard to instrumentation may impact a small fraction of the population of NHPs used in research, minimizing the impact of different constraints may require different solutions. Chipping away constraint-by-constraint may represent a productive approach to reducing the use of single housing.

There was a noticeable decrease in the proportion of facilities reporting clinical exemptions since 2014, but an increase regarding behavioral exemptions (Table 7). Without more details on the nature of these behavioral exemptions, it is difficult to interpret these findings. Care should be taken to ensure that behavioral exemptions are not applied too broadly, and that social housing is employed wherever possible, barring a situation with highly predictable detriment to animal welfare.

The most recent survey found that all facilities experienced practical constraints. This represents an increase since 2014, with most individual/specific constraints being reported more frequently in 2020. The only departure from this pattern was time/staffing, which did not increase, reported as a constraint in a third of facilities at both time points (Table 7). These findings paint a daunting picture, with most constraints creating a headwind against progress in the use of social housing in the US, where NHP facility populations can be large. However, some constraints may be more practical to address than others, and a re-evaluation of the constraints, or perceived constraints, may help establish new processes and standards that can overcome these and make social housing more attainable (see Section 3.1.3 above for discussion on techniques and constraints to social housing).

Notable shifts in introduction techniques since 2014 include decreases in use of initial protected contact, which may make the implementation of introductions less labor intensive. Other decreases were introduction in enclosures larger than primary housing, and medication. In contrast, introduction in neutral enclosures increased (Table 8).

### 3.2. Nursery Rearing

Nursery rearing was defined as purposeful separation of infants from their dams before one year of age. Facilities using nursery weaning gave information on the following issues: the purpose of their nursery rearing, age at weaning, social management practices and the use of single housing for infant NHPs. Not every facility responded to every topic. A subset of respondents (14) reported maintaining NHP nurseries, including 4 EU and 10 US facilities. No UK facilities responded to survey items pertaining to nursery rearing.

**Key findings for nursery rearing are summarized below:**

■ **Prevalence:** Rare in EU; common in US.
■ **Weaning age:** EU facility = nine months; US = birth to six months for some purposes.
■ **Purpose:** Breeding, pathogen-free animals, research subjects.
■ **Key concern:** Early weaning linked to welfare and health risks.

Further details on purpose, age at weaning, and implications for welfare are provided in the following sections.

Regarding the purpose of nursery rearing, response options included ‘with the aim of increasing animal production’ [breeding], ‘deriving pathogen-free animals’, ‘use in research’, and ‘other’. One EU facility and 5 US facilities reported the use of nursery rearing for increasing breeding production (though there is limited literature to support this approach, see discussion below). Five US facilities, but no EU facilities, employed nursery rearing for deriving pathogen-free animals. One EU facility and seven US facilities employed nursery rearing to generate research subjects. One EU facility indicated that nursery rearing was only employed in cases of maternal rejection.

#### 3.2.1. Minimum Age at Weaning

The regulatory approach to weaning varies considerably between regions. The EU Directive and UK Code of Practice require that separation from mothers does not take place until six to twelve months, with specific advice for some species (e.g., not before eight months for marmosets, tamarins, macaques, vervets and baboons, and not before six months for squirrel monkeys). Furthermore, the advice section of the UK Code of Practice stipulates that *“Early weaning from the mother—if it may cause pain, suffering, distress or lasting harm—may be a regulated procedure. In such circumstances the Home Office should be consulted regarding whether Project Licence authority is required.”* In the US, no standards for weaning age are addressed. The only verbiage in the US Animal Welfare Act that is relevant to nursery rearing or weaning, though not explicitly pertaining to it, is the requirement to provide “special attention” to the environmental enhancement for infants and young juveniles.

Eleven facilities reported the minimum age for the removal of infants from dams (1 EU and all 10 US facilities). The age at weaning in the EU facility was nine months regardless of the purpose of nursery rearing, in line with EU regulatory standards. In the US, among all uses of nursery rearing queried, 3 of 10 US facilities weaned at no less than six months (in line with minimum EU standards); 5 reported separation from the dam at birth, and 2 before six months of age. Whilst they are not governed by EU/UK regulations, it is nonetheless notable that 6 US facilities weaned at an age younger than stipulated in these regulations.

Among US facilities, weaning age varied with the purpose of nursery rearing. For increasing breeding production, weaning ranged from birth to one year (with a mean of five months). For generating specific pathogen-free animals, the ages were younger, ranging from birth to seven months (mean three months). For generating research subjects, ages were younger still, ranging from birth to six months (mean two months). The literature pertaining to weaning age in NHPs is surprisingly sparse. With respect to rearing in the context of breeding colonies, findings are variable as to whether early weaning results in higher levels of production in comparison to weaning at a more species-appropriate age with recent data not supporting an increase in productivity [80,81]. Apart from this concern, early weaning predisposes macaques increased *Campylobacter* infection, which can interfere with NHPs use in breeding colonies, and increased alopecia prevalence and severity [80]. However, one should not lose sight of the fact that behavioral competence for successful reproduction and rearing of offspring is best supported by avoiding nursery rearing altogether [38,82].

While information regarding age at weaning is of great relevance to support welfare, the small sample size of survey respondents precludes the assessment of patterns associated with the purpose of nursery rearing, regional differences, and the intersection of these factors. The lower weaning age in the US, where there is a lack of specific advice compared to other regions, demonstrates the benefit of having clear guidance on this topic to ensure best practice. Regardless of their location, NHP facilities should review weaning ages regularly to ensure that practices are as supportive of welfare as possible and fully justified.

#### 3.2.2. Use of Single Housing for Nursery Rearing

Single housing in the nursery was used in half (2) of the EU facilities and in all 10 US facilities, and generally for more than one reason. Half of the facilities (1 EU and 5 US facilities) reported using single housing for ‘approved research protocol justifications.’ One EU facility and 9 US facilities employed nursery rearing due to severe medical conditions or maternal abuse/incompetence. Five US (but no EU) facilities reported delaying the onset of social housing as a matter of management policy, for example while infants are in incubators, or in an attempt to curb clinging. Two US facilities reported singly housed infants due to the unavailability of social partners.

#### 3.2.3. Social Housing Practices for Nursery Rearing

There are no specific regulatory stipulations for the manner of providing social housing for nursery rearing. Three relatively common methods were queried in the survey: (i) peer rearing with continuous social housing with a stable set of partners; (ii) peer rearing with continuous social housing but with the rotation of different partners; or (iii) surrogate peer rearing (continuous access to an inanimate surrogate with some social housing via access to peers for part of the day). Responses were provided for 2 of the 4 EU facilities and all 10 US facilities, with most facilities using more than one of these social housing practices. Peer rearing with continuous access and consistent partners was reported at the largest proportion of facilities, including the 2 reporting EU facilities and all 10 US facilities. Continuous social housing with rotating partners was used in 1 EU and 3 US facilities, whereas surrogate peer rearing was reported in 1 EU and 5 US facilities.

Of these strategies, whilst no approach has been deemed superior, each show differing effects depending on age, context (stressed vs. stable), and domains (e.g., abnormal behavior, social behavior, stress response, health measures). Supportive literature is too vast to fall within the scope of this paper and many papers do not compare all three strategies. However, the purpose for which nursery-reared individuals are produced may guide the methods used for social housing in the nursery. For example, generating infants for research may be a purpose for which continuous peer housing leads to better outcomes than surrogate peer housing in terms of predisposing individuals to the development of serious behavioral abnormalities [83,84,85,86] which pose welfare challenges and can interfere with research use. This may be particularly important where the research procedures themselves are also risk factors for the development of behavioral pathologies [84].

Where rearing may be a confound to research using subjects of varying backgrounds, both surrogate-peer rearing and housing in rotating pairs may be beneficial in dampening differences between mother- and nursery-reared individuals [85,87,88]. When infants are nursery reared and will be integrated into a breeding setting, surrogate-peer rearing may be most appropriate in that it results in generally more normal social behavior [88,89,90,91,92]. This is thought to relate to the discouragement of clinging to peers, because when singly housed infants cling to inanimate surrogates they are then more likely to interact normally with peers in the short periods with access to them.

Across all nursery rearing purposes, decisions need to be made with a full appreciation of the perturbations caused by nursery rearing across numerous domains (see [87,93,94,95,96] for reviews and example studies on macaques; also see [97,98,99,100] for examples of results for other NHP species). These perturbations may compromise the value of research and contribute to significant detriments to welfare. Refinements to nursery rearing are therefore important to reduce physiological and welfare impacts.

### 3.3. Enclosures

The dimensions and features of enclosures that maximally support NHP psychological well-being depend on factors such as species’ body size, social organization, and ethology [101,102]. NHPs may be housed in a range of enclosure types (including indoor runs, cages and outdoor enclosures), which vary in purpose, size and features they incorporate. Therefore, animal management can differ between housing types. Further information is given in [12,103] and the NC3Rs macaque website (https://macaques.nc3rs.org.uk).

**Key findings for enclosures are summarized below:**

■ **Space provision:** 46% of facilities exceeded minimum floor space for all NHPs; EU/UK (83%), US (21%).
■ **EU-style pens:** Used by 56% of facilities (UK 100%, EU 83%, US 37%).
■ **Exercise enclosures:** Used by 55% overall; most common in US (67%); daily access in 67% UK and 41% of US facilities. EU facilities reported access several times a week rather than daily.
■ **Top constraints:** Space limitations and cost.
■ **US trend:** Exercise enclosure use increased (50% → 67%).

Further details on enclosure size, EU-style pens, and exercise enclosures are described in the following sections.

Conventional cages are typically fashioned from stainless steel bars, mesh, and solid panels, and installed on metal rolling racks, with dimensions designed to meet regulatory minimums for cage space and suitability for commercially available cage washers. They are historically used for indoor single housing for biomedical research, but more recently they are being used to provide social housing via linked cages or cage units. Indoor pens and enclosures with or without access to the outdoors are intended for social groups and may have a variety of floorings such as natural substrates, gravel, or solid surfaces. ‘European-style’ or ‘EU-style’ pens are enclosures that meet the EU Directive 2010/63/EU requirements [16] and include features such as visual barriers for greater control over social interactions, solid flooring to facilitate the use of substrates for extended hours of foraging behavior, ample means for climbing and locomotion, ‘porches’ extending out to provide better visibility into the room, and what is judged to be sufficient three-dimensional space to retreat from humans. EU-style pens are generally seen as an improvement over small conventional cages at or near minimum legal sizes.

Facilities were queried about enclosure space, the size of the primary enclosure, and the use of EU-style pens. The survey also queried the use of exercise enclosures, which are caging or pens that are not part of the primary enclosure but provide greater space. Facilities were also asked about constraints to the implementation of exercise enclosures. Forty-six facilities responded to at least some of the questions regarding enclosures (12 EU, 6 UK, and 28 US).

#### 3.3.1. Enclosure Size

Whilst there is variation between NHP species across multiple parameters, including size, social organization, and behavior, regulations tend to stipulate minimum dimensions based upon typical species weights and/or age. These minimums vary across regions (Table 9 and Table 10) with the same trends for different species and ages. The EU Directive and UK Code of Practice require that an individual macaque (or pair) over the age three years should be housed in an enclosure with a minimum volume of 3.6 m^3^, whereas US regulations require a minimum of 0.30 m^3^ per NHP up to 10 kg, which is more than ten times smaller than the European standards.

Facilities were asked whether animals were housed in enclosures that provided more than the minimum applicable regulatory/accreditation floor space for the relevant region, as outlined in Table 9 and Table 10. All facilities reported that at least some (>1%) of their NHP populations were housed in enclosures exceeding the applicable regulatory/accreditation minimum floor space in their region, with 46% of facilities (EU: 83%, UK: 83%, US: 21%) providing more than the minimum floor space to their entire NHP population. However, it should be noted that exceeding the minimum in the US could still mean that NHPs are housed in enclosures that are smaller than EU requirements. It is notable that far fewer US institutions provided more than the minimum floor space to all NHPs, compared to the EU and UK institutions, where minimum sizes are already higher (Table 9 and Table 10). These differences undoubtedly relate to the fact that regulations regarding enclosures for NHPs in the US, aside from the sizes outlined in Table 9, have only a single and minimal performance standard: “*the individual must have the ability to make normal postural adjustments with freedom of movement.*” In contrast, EU and UK regulations incorporate detailed performance standards, namely “*The environment shall enable non-human primates to carry out a complex daily programme of activity. The enclosure shall allow non-human primates to adopt as wide a behavioural repertoire as possible, provide it with a sense of security, and a suitably complex environment to allow the animal to run, walk, climb and jump*.”

Furthermore, the advice section of the UK Code of Practice emphasizes the need for height, particularly for marmoset and tamarin housing: “*In contrast to non-arboreal mammals, the flight reaction of NHPs from terrestrial predators is vertical, rather than horizontal; even the least arboreal species seek refuge in trees or on cliff faces. As a result, consideration should be given to an adequate enclosure height that allows the animal to perch at a sufficiently high level for it to feel secure*” and “*Enclosures should be of adequate height to allow the animal to flee vertically and sit on a perch or a shelf, without its tail contacting the floor or head touching the roof of the cage; enclosures should not be arranged in two or more tiers vertically.*” Particularly in taxa other than callitrichids, two-tiered caging is common practice in the US.

Both the lower minimum cage sizes and the absence of ambitious performance standards in the US likely contribute to these regional contrasts. While there have been a number of studies on the association between three-dimensional cage size and welfare, its significance is reduced in many cases due to other factors that contribute to NHP welfare [104]. However, studies involving differences in cage height have shown benefits to animal welfare [105,106,107,108]. More research is required for data-driven decisions regarding increases in enclosure dimensions.

#### 3.3.2. ‘European-Style’ Pens

Over half (56%) of facilities reported using EU-style pens (EU: 83%, UK: 100%, US: 37%), with 46% of these facilities (EU: 58%, UK: 100%, US: 0%) providing them to their entire population. Whilst this style of housing is not a requirement in the US, it is encouraging that 37% of US facilities provide EU-style pens to some of their animals, as they are associated with significantly higher performance standards. In the US, the relationship between the use of EU-style pens and the proportion of animals provided more than the minimum amount of space suggests that the pens are playing some role in providing extra space; 60% of facilities providing extra space to all individuals used them, compared to 32% of facilities that did not. However, there are numerous other ways to provide extra space, including the use of enclosures other than caging, or linking cages together vertically or horizontally which is necessary for the provision of functionally appropriate climbing substrates and may be more significant to welfare than floor space. When accompanied by an increase in the use of social housing, these steps may not always reduce cage capacity/stocking density nor incur major financial cost.

#### 3.3.3. Exercise Enclosures

Exercise enclosures consist of caging or pens that are not part of the primary enclosure but provide greater space to permit more locomotion, activity and enrichment. There are no regulations pertinent to this form of enhancement, but the UK Code of Practice (advice section) mentions it only as a mechanism for short-term confinement (e.g., whilst moving animals) rather than as an expansion of space.

Across all facilities, exercise enclosures were used for caged NHPs at 55% of facilities (EU: 27%, UK: 50%; US: 67%). Among these facilities, exercise enclosures were distributed to all caged animals at facilities in the EU and UK (67% and 100%, respectively). The NHP guidelines of the NC3Rs, adopted by other UK research funding bodies, mandate their use for caged animals [18]. However, in the US, only 1 facility provided exercise enclosures to all caged NHPs. The need for an exercise enclosure may depend on the size and type of enclosure in which NHPs are housed. Exercise enclosures are typically used for NHPs housed in cages as opposed to enclosure types that are larger, such as EU-style pens and/or outdoor enclosures. Thirty-nine percent of facilities housing NHPs in EU-style pens used exercise enclosures, compared to 68% of facilities without EU-style pens. Among institutions housing all NHPs in EU-style pens, 20% also used exercise enclosures, while 58% of facilities that housed only a portion of their population in EU-style pens used exercise enclosures as well. These contrasts indicate to some degree that the EU-style pens can obviate the need for exercise enclosures.

Further information on the populations or purpose for utilizing exercise enclosures was provided only by US facilities: intervention for undesirable behavior (44%), to provide more exercise to combat obesity (39%), for singly housed NHPs (33%), for clinical needs (28%), relating to age (young animals) (22%), and scientific requirement (22%).

Information regarding the frequency of placement in an exercise enclosure was reported by 22 facilities (2 EU, 3 UK, and 17 US), ranging from less than once a month up to daily. In the UK and US, the most commonly cited schedule was daily, whereas the only schedule cited in the EU was several times per week (Table 11). The US was the only region that reported the use of exercise enclosures less than once a week or once a month.

Information on the duration of time spent in the exercise enclosure (a full day or less than a full day) was reported by 11 facilities: 1 facility each in the EU and UK and 9 in the US. Animals did not remain in the exercise enclosure for the full day at the EU facility but did so in the one reporting UK facility and in 78% of US facilities.

There is limited literature to guide schedules for the use of exercise enclosures. One study evaluated behavior during a two-week period of continuous exercise enclosure housing. It found several positive behavioral changes, but no carry-over effect after return to standard caging [109]. Given the wide variety of potential schedules, further research is needed to guide best practice, particularly for US facilities where access to exercise enclosures is more limited.

Facilities were asked about constraints preventing the use of exercise enclosures. In each region, available space for exercise enclosures was the most cited reason, and the only constraint identified in any of the UK facilities (Table 12). The cost of exercise enclosures was also a significant constraint in the EU and US. Only US institutions cited constraints involving time/staffing, concerns over negative consequences, and research requirements. No facilities stated that a belief about lack of benefit constrained the use of exercise enclosures, suggesting its acceptance as best practice. In the free text, two US facilities cited concerns for personnel safety and one the challenge of effectively removing individuals from large enclosures and performing this process in a safe manner for staff. Practical guidance on managing NHPs in similar husbandry systems is available in the literature [103,110] or can be sought from other institutions.

#### 3.3.4. Changes to the Use of Exercise Enclosures in US Facilities

The use of exercise enclosures was not queried in 2003. Since 2014, the proportion of US facilities employing exercise enclosures has increased (from 50% in 2014 to 67% in 2020), also shifting its most common schedule (from once a week or once a month) to daily (Table 13). However, distribution to all of their population fell among facilities providing exercise enclosures, from 32% to 8%. Most of the reported constraints on exercise enclosures reported in 2014 have decreased over time, except for concern over negative consequences or cost, which have remained the same (Table 14). In 2020, no facility expressed the belief that exercise enclosures conferred no benefits to well-being, which is concordant with their increased use. In contrast to social housing for which reports of scientific constraints increased over time, scientific exemptions to the use of exercise enclosures were negligible.

### 3.4. Destructible Enrichment

Destructible enrichments were divided into two categories in the survey: (1) flooring substrate and bedding material, which we term here as ‘Substrates’ and (2) readily mutable material including paper, cardboard, nondurable plastic, and cloth, which we term ‘other destructibles’.

**Key findings for destructible enrichment are summarized below:**

■ **Overall use:** For caged NHPs, substrates were used by 61% of facilities (EU 90%, UK 100%, US 38%), and other destructibles were provided by 90% of facilities (EU and UK: 100%; US: 85%).
■ **Top constraints:** Plumbing/drainage issues; sanitation concerns.
■ **US trend:** Use increased since 2003; constraints decreased slightly.

Further details on implementation levels, constraints, and opportunities for refinement are described in the following sections.

We differentiated destructibles into substrates and other destructibles because they may be employed for different purposes and because EU and UK regulations address bedding/forage material but not other destructible enrichment. There are no regulations specific to destructible enrichment as a broad category. Regulations in the EU and UK include requirements for bedding and material for nesting and foraging. In addition, the advice in the UK Code of Practice recommends that rhesus macaques and vervets be housed on solid floored pens because these species spend considerable time on the ground. It also states: “*Solid floors have the advantage that they can be covered with a substrate in which food can be scattered to encourage foraging*” and “*Where grid floors are used, the animals should have access to a suitable solid resting and foraging area.*” The USDA regulations do not require the provision of flooring/bedding nor the use of flooring that would render substrates feasible. The only text relevant to destructible enrichment of any kind consists of mention of the use of foraging amongst a list of examples of environmental enrichments. Destructible enrichment can also provide sustained novelty, as it is altered by use.

Forty-six facilities responded to questions about destructible enrichment (12 EU, 6 UK, and 28 US), which queried implementation and constraints to use. For information regarding implementation levels, the survey asked separately about implementation for caged NHPs and those housed in other enclosure types.

Levels of Substrate use varied between enclosure type. For caged NHPs, 61% provided Substrates (EU: 90%, UK: 100%, US: 38%), and among these facilities, substrates were provided to all caged NHPs in 64% (EU: 78%, UK: 100%, US: 25%). In other types of enclosures, implementation levels were higher; 78% of NHPs received Substrates (EU: 100%, UK: 100%, US: 69%), with 56% of these facilities providing it to all NHPs (EU: 67%, UK: 100%, US: 45%).

Similarly, the use of other destructibles varied between housing types. For caged NHPs, 90% provided other destructibles (EU: 100%, UK: 100%, US: 85%), and among these facilities, other destructibles were provided to all caged NHPs in 64% (EU: 90%, UK: 100%, US: 59%). As with Substrates, implementation levels for other destructibles were slightly higher for NHPs in other types of enclosures, with 79% of facilities providing them (EU: 100%, UK: 100%; US: 65%). Among these facilities, all NHPs received other destructibles at 74% of facilities (EU: 67%, UK: 100%, US: 64%).

#### 3.4.1. Constraints on the Use of Destructibles

The survey did not differentiate between Substrates and other destructibles when querying constraints. UK institutions provided destructibles to all animals in all caging types and unsurprisingly reported no constraints to its implementation. For both the EU and US respondents, plumbing (i.e., clogging of pipes) was the most commonly cited constraint, followed by reasons related to time/staffing and other impacts on sanitation (Table 15). It should be noted that the use of flooring substrates may be problematic for indoor metal cages with grate flooring, although this was not specifically noted as a constraint in this survey. In free text, several facilities commented that their NHPs were housed outdoors on natural ground cover, so destructibles were not considered to be necessary.

Flooring substrates have been studied in a number of different laboratory NHP species and are associated with a variety of positive behavioral changes [111,112,113,114,115,116]. Major constraints on the use of destructibles have been measured in several studies, which focused on flooring substrates, but have implications for many types of destructibles. These papers have quantified changes in labor and other costs and offered engineering solutions to curb the impact on plumbing, with favorable findings [115,117]. Studies of other sorts of destructibles appear to be absent from the peer-reviewed literature. Using water-soluble paper for caged NHPs would also avoid plumbing issues. Identifying and quantifying practical constraints through future research, and disseminating the findings via conferences and other publications, will help drive innovative solutions that enable broader implementation of destructibles, including flooring substrates.

#### 3.4.2. Changes in the Use of Destructibles Within US Facilities over Time

The 2003 and 2015 survey did not differentiate between Substrates and other destructibles, so 2020 values collapse the two categories. In addition, the 2003 survey did not distinguish implementation in cages vs. other enclosures, but they were distinguished in the later surveys. The use of destructibles in US facilities has increased over time. In 2003, their use was reported at 59% of facilities. In 2014, use increased for both enclosure types (caging: 85%; other enclosures: 76%), but this remained unchanged in 2020 (caging: 89%; other enclosure types, 71%).

Constraints were not queried in 2003. Most constraints were reported by a smaller proportion of facilities in 2020 than in 2014, in line with the increased use of destructibles, though there was an increase in the reporting of scientific exemptions to the use of destructibles in 2020 compared to 2014 (Table 16). The most commonly cited constraint (drainage/plumbing) was reported at the same level in both years. Despite its ubiquitous use in the UK, this challenge appears yet to be resolved in the US.

### 3.5. Sensory Enrichment

Facilities were queried as to the use of sensory enrichment techniques, described in the survey questionnaire as passive enrichment such as music, video, or aromas. None of the regional regulations include standards for sensory enrichment and there are few studies guiding the implementation of this form of enrichment, so practices are often based on experience.

**Key findings for sensory enrichment are summarized below:**

■ **Overall use:** Implemented in 90% of facilities for caged NHPs (64% provided to all animals); 52% for other enclosure types (54% provided to all animals).
■ **Top constraints:** Cost and staffing; some facilities (12%) questioned welfare benefits.
■ **US trend:** Use stable since 2014; constraints (cost/time) increased.

Further details on current practices, constraints, and evidence gaps are provided in the following sections.

Forty-three facilities provided information (11 EU, 6 UK, and 26 US). Where provided to caged NHPs (All regions: 90%, EU: 90%, UK: 80%, US: 92%), all caged NHPs at a facility were provided sensory enrichment at 64% of facilities (EU: 78%, UK: 75%, US: 58%). Sensory enrichment was provided to NHPs housed in other enclosures at 52% of facilities (EU: 80%, UK: 33%, US: 47%). Where used, it was provided to all NHPs at 54% of facilities (EU: 50%, UK: 100%, US: 50%).

#### 3.5.1. Constraints to Use of Sensory Enrichments

Cost and time/staffing were the most prevalent constraints on the provision of sensory enrichment reported across all institutions (Table 17). Several institutions noted that their NHPs were housed outdoors and therefore subject to a variety of scents, so this form of enrichment was not considered a necessary provision. Unlike all the other components of behavioral management reported here, no facilities reported scientific (research protocol) exemptions. However, this was the only aspect of behavioral management where a belief in a lack of benefit was noted. This is somewhat consistent with the literature, which provides relatively little guidance for determining the benefits of sensory enrichment to NHPs. Results are inconsistent with negative effects seen in some [118,119,120]. Some studies find no effects [121]. Others suggest benefits but are limited in implication due to their anecdotal reports, their examining a very short-term period of time, measuring only the context of brief presentations of sensory enrichment, or studying sensory enrichment in association with operant conditioning [122,123,124,125,126]. The ability to draw conclusions from the available literature is also constrained by the wide variety of sensory enrichments evaluated.

This paucity of literature, especially in laboratory settings, does not necessarily suggest a lack of benefit [22,127]. Some literature suggests there may be benefits for some forms of sensory enrichment with specific functions (i.e., rather than looking for sustained improvements in indices of welfare). For example, providing the opportunity to view potential pair partners via video conferencing technology may be useful for guiding social management [128,129]. Adding sensory enrichment in the form of neutral noise has been used to mask potentially stress-inducing sound (conspecific or anthropogenic) for species that may be particularly vulnerable to it [130,131]. Some forms of sensory enrichment provide opportunities for exerting choice and control, and this ability has been found to be associated with behavioral and welfare benefits [125,132]. With respect to the most commonly reported constraints (i.e., cost), sensory enrichment may encompass a wide variety of techniques associated with different levels of expense. Thus, there should be opportunities for laboratory facilities to add or expand the provision of more diverse sensory stimuli to NHPs through a number of techniques, especially those with a relatively low cost.

#### 3.5.2. Changes in the Use of Sensory Enrichments Within US Facilities over Time

Sensory enrichments were not queried in 2003. In both 2014 and 2020, the proportion of facilities that used sensory enrichment remained steady, both for caged NHPs (2014: 90%; 2020: 92%) and those housed in other enclosure types (2014: 50%; 2020: 47%). Scientific exemptions were no longer reported in 2020 (Table 18). Reports of constraints related to time/staffing and cost increased from 22% in 2014 to 47% in 2020. The costs and time investments associated with different forms of sensory enrichment vary dramatically, and the inclusion of more feasible options could boost levels of use.

### 3.6. Cognitive Enrichment

The survey queried the use of cognitive enrichment, defined as techniques that aim to provide challenging tasks to engage intelligence to solve problems (e.g., computer games and puzzle feeders). The EU Directive, UK Code of Practice and NC3Rs guidelines require that facilities employ enrichment techniques that extend an individual’s range of activities including physical exercise, foraging, manipulative and cognitive activities. However, requirements for cognitive activities are not addressed in the US Animal Welfare Act.

**Key findings for cognitive enrichment are summarized below:**

■ **Overall use:** Implemented in 91% of facilities for caged NHPs; all animals at 46% of those facilities. For other enclosure types, implementation was lower (57%), with 54% of facilities providing to all NHPs.
■ **Top constraints:** Time and cost; scientific exemptions rare (reported only by US facilities at 12%).

Further details on implementation levels, practical constraints, and emerging technologies are described in the following sections.

Forty-three facilities (11 EU, 6 UK, and 26 US) provided information on the use of cognitive enrichment. For use with caged NHPs, levels were high in all regions (All regions: 91%, EU: 91%; UK: 83%, US: 92%). Among facilities implementing cognitive enrichment, it was provided to all caged NHPs at 46% of facilities (EU: 44%, UK: 83%, US: 33%). With the exception of the UK, levels of cognitive enrichment for NHPs housed in other types of enclosures were lower than those for NHPs housed in cages (All regions: 57%, EU: 80%, UK: 100%, US: 47%). Among these facilities, 54% of facilities provided it to all NHPs (EU: 50%, UK: 100%, US: 50%). Contrasts between housing types may relate to the view that enclosures other than cages provide greater opportunities for the expression of cognitive activity, due to their higher level of social and inanimate complexity.

#### Constraints to Use of Cognitive Enrichments

Across all facilities, constraints on cognitive enrichment were largely related to time/staffing and cost (Table 19). Only US institutions reported that scientific exemptions impacted the implementation of cognitive enrichment, and only one EU institution reported concerns regarding negative consequences or skepticism regarding benefits. This demonstrates that the value of cognitive enrichment for NHPs is recognized, but provision may not always be possible due to practical or economic constraints.

Enrichment devices, especially those that pose some cognitive challenge (e.g., puzzles) rather than merely extractive challenges, may be costly to adopt, particularly at large institutions. Access to higher-level challenges such as those provided by computer games are even more financially daunting. Many neuroscience experiments with NHPs require the animals to perform extensive and challenging cognitive tasks with escalating difficulty. Such studies use sophisticated computerized training and testing equipment that can be adapted to provide an extensive level of cognitive enrichment. Traditional systems can entail considerable effort in transporting animals to/from the apparatuses and protecting the apparatuses from damage (e.g., caused by the animals or, if maintained outdoors, inclement weather). For socially housed NHPs, additional challenges can include enabling access for all group members, individual identification and identifying an appropriate level of challenge, further increasing the cost associated with microchipping or facial recognition cameras.

Recently, however, a variety of home cage, touch-screen, automated training systems have been developed which are simpler and cheaper to buy/create and maintain [59,133,134,135]. NHPs will engage with these cage-based systems for long periods and appear to find this inherently rewarding without food or fluid control. These relatively cost-effective, more easily implemented alternatives could increase the provision of cognitive enrichment and enable further studies of this aspect of behavioral management.

Many studies evaluating the use of computerized enrichment use engagement as a metric relating to improved welfare [136] without other measures. The utility of the literature is also hampered by small numbers of subjects. In addition, they may evaluate welfare only in the presence of the equipment and with continuous access (which is unlikely to be tenable in most circumstances), and/or report changes in behavior that do not relate to welfare [126,137,138,139]. Studies that comparing welfare with and without the presence of the equipment, as well as comparing equipment of various costs, would be valuable for expanding the use of cognitive enrichment [140].

## 4. Discussion

The following discussion expands on the implications of these findings for best practice and future research. This study relied on convenience sampling and voluntary participation, a technique which limits generalizability and introduces potential response bias. While this approach is common in welfare surveys and allowed broad international participation, findings should be interpreted as indicative rather than representative of all facilities globally.

The findings of the survey only represent the practices at the time the survey was conducted, and not all regions were represented equally. However, practices in a sufficient number of institutions were captured in the EU, UK and US to illustrate variation in NHP behavioral management across these regions. There were not enough responses from elsewhere in the world to make similar deductions in other regions, information useful to pursue in a future survey. A wider awareness of the variability of behavioral management practices can help in evaluating programs and to provide an expanded view of what is feasible when caring for NHPs in the laboratory environment, with the aim of harmonizing best practices more globally.

Responses from facilities in the EU and UK suggest that they are clearly at the forefront of best practice in many aspects, including the proportion of NHP populations housed socially, greater age at which infants may be removed from dams, and provision of pen enclosures, as well as the level of implementation of caging exceeding the regulatory/accreditation minimum floor space/cage volume, floor substrates and other destructible enrichment. This may reflect the higher standards/requirements for supporting the welfare of animals used for scientific purposes and the strength of the oversight framework in the UK and EU compared to the US. Several of the more stringent requirements in the UK and EU compared to the US correspond directly to survey findings, including minimum cage size, social housing and age of weaning. The lower weaning age in the US, where unlike other regions, there is a lack of specific advice or regulatory requirements demonstrates the benefit of having clear guidance on this topic to ensure best practice. However, there were also differences in aspects not covered in regulations, with many UK and EU institutions exceeding minimum space allocations and using additional enrichments such as destructibles, which may demonstrate the influence of ambitious performance standards across all areas of behavioral management, driving expectations and elevating the culture of care. Key to driving improvements in the aforementioned aspects of behavioral management were the decisions of the UK’s NHP-research funding bodies to specify their own guidelines for NHP accommodation, care and use, led by the NC3Rs in 2006 and 2017, and to make implementation of these high standards a condition of grant funding for studies conducted elsewhere in the world [18]. Compliance with the guidelines is assessed as part of the peer review process for research grant applications. At the time of writing, the ILAR *Guide for the Care and Use of Laboratory Animals* is under revision. The Guide is used as an accreditation standard for evaluation of NHP care and use programs, and facilities must meet these standards to obtain grant funding from the National Institutes of Health. Modifications to the Guide will be a mechanism for promoting change and elevating expectations for NHP care, as they have been in the past.

In the US, reasons for not providing social housing included practical constraints such as costs, time/staffing, housing type, space, or delays associated with obtaining necessary information from investigators—constraints that were not reported as commonly in other regions. This finding, as well as some other behavioral management techniques, may reflect, amongst other reasons, the typically larger facility sizes in the US compared to the other regions (the US facilities captured in this survey are typically 8 and 16 times larger than EU and UK facilities, respectively, with EU facilities typically twice as large as UK facilities). Implementing change in larger institutions has a much larger initial financial and resource commitment, which may be prohibitive in some cases. Whilst the UK is at the forefront of best practice in many aspects, these facilities are generally small in comparison to those in the US (of facilities responding to the survey, UK institutions housed a maximum of 500 NHPs, compared to 999 in the EU and 7571 in US facilities), and so constraints related to cost may not be as much of an impediment.

However, positive changes and progress are being made over time in the US, and in comparison to the previous surveys in 2003 and 2014 there were a greater proportion of facilities providing access to exercise enclosures, more often on a daily basis. In addition, the provision of EU-style pens increased. However, progress in the use of social housing was limited, with no increase over the prior six years despite its widely recognized welfare benefits. There was, however, some improvement in the use of social housing for some of the species reported in the survey.

In all regions, scientists and all those involved in the care of the animals may benefit from learning more about the strategies used to accommodate social housing, to help overcome constraints and make this form of housing more widely feasible within their institution. This also applies to the other forms of behavioral management covered in the survey. Opportunities for knowledge transfer include events and platforms such as conferences, meetings and online resources such as the NC3Rs macaque website (https://macaques.nc3rs.org.uk) which provides practical advice on overcoming constraints and implementing best practices. Other resources include the welfare material presented on the website of American Society of Primatologists, annual conferences focusing on NHP behavioral management or with heavy emphasis on it (e.g., the American Association for Laboratory Animal Science, American Society of Primatologists), and workshops (Primate Behavioral Management Conference and the Workshop of Macaque Pair Housing), NHP welfare assessment tools and published outcomes are another means for facilities to objectively evaluate numerous elements of their own programs, identify areas deviating from best practices and requiring focus for program improvement [71,141,142].

Here we have used the survey results and the available literature to make recommendations relating to social housing, nursery rearing, enclosure sizes, and use of destructible enrichment, floor substrates, sensory and cognitive enrichment. Other aspects of behavioral management such as positive reinforcement training, positive human interaction, management of behavioral pathology, program administration, and employee training were also queried in the survey but are not included in this manuscript. Nonetheless, these areas represent additional opportunities to improve practices and harmonize the care and use of laboratory animals.

It is clear that comprehensive behavioral management programs are fundamental to ensure good welfare in captive NHPs. Key to improving programs are regulatory standards and guidelines along with appropriate mechanisms for oversight and enforcement, as well as an understanding of the value and need for strong behavioral management practices. With knowledge transfer and sufficient resources, constraints on key elements of behavioral management can be overcome to improve NHP welfare and facilitate good science. Investment into behavioral management research is important to increase scientific knowledge to guide programs. As scientific evidence continually expands and evolves, there will be a need to regularly review current practices and share approaches across all regions to understand and promote best practice. There would therefore be benefit in periodically repeating the survey and widening the participation outside of the US, UK and EU, into regions in which standards and approaches may not be so high, to improve the care and use of laboratory animals and ensure higher standards worldwide.

## 5. Conclusions

This study successfully achieved its primary objective: to analyze and compare behavioral management practices for laboratory-housed nonhuman primates across the EU, UK, and US. It provides the most comprehensive international snapshot to date, revealing substantial regional differences, persistent constraints, and long-term trends. These findings underscore the critical role of robust behavioral management in improving NHP welfare and scientific outcomes and identify clear opportunities for refinement and harmonization across regions. By sharing these insights and practical recommendations, we aim to support institutions in benchmarking their programs and adopting best practices. Future progress will depend on continued knowledge exchange, expanding participation to other regions, investment in behavioral management research, and periodic global surveys to track change. Strengthening behavioral management worldwide is essential to uphold high ethical standards, advance the 3Rs, and ensure the quality and reproducibility of science.

## Figures and Tables

**Table 1:** Number of facilities housing each species.

Species	No. Facilities			
	All Regions	EU	UK	US
Rhesus macaque	34	9	5	20
Long-tailed macaque	32	7	2	23
Common marmoset	11	5	0	6
Pig-tailed macaque	8	1	0	7
African green/vervet monkey	7	2	0	5
Common squirrel monkey	6	1	0	5
Baboons	5	1	0	4
Capuchins	5	2	0	3
Owl monkeys	2	0	0	2
Mangabeys	2	0	0	2
Japanese macaque	1	0	0	1
Titi monkeys	1	0	0	1
Other species	5	2	1	2

**Table 2:** Extracts from relevant regulations regarding the use of social housing.

**The EU Directive 2010/63/EU, Annex III:** ■ “Animals, except those which are naturally solitary, shall be socially housed in stable groups of compatible individuals. In cases where single housing is allowed in accordance with article 33(3) the duration shall be limited to the minimum period necessary and visual, auditory, olfactory and/or tactile contact shall be maintained. The introduction or re-introduction of animals to established groups shall be carefully monitored to avoid problems of incompatibility and disrupted social relationships.” ■ “States may allow exemptions from the requirements of paragraph 1(a) or paragraph 2 for scientific, animal-welfare or animal-health reasons.” ■ The tables on space allocations for NHPs within the Annex (6.1 to 6.3) states: “Animals shall be kept singly only in exceptional circumstances.”
**The UK Code of Practice for the Housing and Care of Animals Bred, Supplied, or Used for Scientific Purposes (2014)**: ■ “Animals, except those which are naturally solitary, should be socially housed in stable groups of compatible individuals. Single housing should only occur if there is justification on veterinary or welfare grounds. Single housing on experimental grounds should be determined in consultation with the animal technician and with the competent person charged with advisory duties in relation to the well-being of the animals. In such circumstances, additional resources should be targeted to the welfare and care of these animals. In such cases, the duration should be limited to the minimum period necessary and, where possible, visual, auditory, olfactory and tactile contact should be maintained.”
**The USDA Animal Welfare Act**: ■ “Social grouping. The environment enhancement plan must include specific provisions to address the social needs of nonhuman primates of species known to exist in social groups in nature. Such specific provisions must be in accordance with currently accepted professional standards, as cited in appropriate professional journals or reference guides, and as directed by the attending veterinarian. The plan may provide for the following exceptions:(1) If a nonhuman primate exhibits vicious or overly aggressive behavior, or is debilitated as a result of age or other conditions (e.g., arthritis), it should be housed separately; (2) Nonhuman primates that have or are suspected of having a contagious disease must be isolated from healthy animals in the colony as directed by the attending veterinarian.” ■ “For a research facility, the Committee may exempt an individual nonhuman primate from participation in some or all of the otherwise required environment enhancement plans for scientific reasons set forth in the research proposal. The basis of the exemption shall be documented in the approved proposal and must be reviewed at appropriate intervals as determined by the Committee, but not less than annually. The attending veterinarian may exempt an individual nonhuman primate from participation in the environment enhancement plan because of its health or condition, or in consideration of its well-being. The basis for exemption must be recorded by the attending veterinarian for each exempted nonhuman primate.”

**Table 3:** Percentage of animals of each species housed socially across all housing types.

	All Regions	EU	UK	US
**All species**	81	93	96	80
Common squirrel monkey	99	100		99
Baboons	96	99		96
Capuchins	93	100		78
Common marmoset	83	93		78
Long-tailed macaque	80	94	100	77
Rhesus macaque	80	81	62	80
African green/vervet monkey	73	100		73
Japanese macaque				99
Titi monkeys				98
Owl monkeys				88
Mangabeys				87
Pig-tailed macaque				78

Note: Cells in the ‘All regions’ column are blank where species were reported only in one region. Other cells are blank for regions in which a species was not housed.

**Table 4:** Techniques associated with introduction of NHPs for social housing, by region (percent of facilities).

Techniques	Percent of Facilities			
	All Regions	EU	UK	US
Use of compatibility/hierarchy data from supplier	77	69	57	86
Initial phase of visual access	77	77	57	82
Protected contact prior to full contact	77	85	57	79
Observation of affiliation required to deem introduction successful	65	92	57	54
Introduction in neutral cages unfamiliar to all potential partners	52	46	29	61
Introduction of related animals when possible	31	23	14	39
Group formation from subgroups	29	31	29	30
Contraception	29	31	14	33
Introduction of large groups in neutral enclosures	29	31	14	32
Introduction in enclosures larger than primary housing	21	31	0	21
Canine blunting	19	23	14	19
Introduction in enclosures smaller than primary housing	10	8	14	11
Medication	10	8	0	14

**Table 5:** Rationales for the use of single housing: Behavioral/welfare, clinical, and scientific exemptions, and practical constraints (percent of facilities). The number of facilities responding regarding specific scientific exemptions are provided in parentheses because responses presented here are drawn only from facilities reporting the relevance of each exemption, which varied significantly in number.

Constraints	% Facilities (no. Facilities Responded)			
	All Regions	EU	UK	US
**Behavioral/welfare exemption**	65	36	100	73
**Clinical exemption**	31	14	33	37
**All scientific exemptions**	69	60	33	83
Concerns about cross-infection	64 (22)	50 (4)	0 (2)	75 (16)
Urine collection	60 (20)	60 (5)	100 (2)	54 (13)
Tethering	56 (16)	33 (3)		62 (13)
Measure food/fluid consumption	50 (24)	17 (6)	0 (1)	65 (17)
Anticipated rapid decline	48 (23)	40 (5)	0 (1)	53 (17)
Restricted/controlled diets (food or fluid)	30 (27)	17 (6)	0 (3)	39 (18)
Internal implants (e.g., in the body cavity, vascular access ports)	9 (23)	14 (7)		6 (16)
Jackets	6 (17)	25 (4)		0 (13)
Cranial implants	4 (24)	0 (7)	0 (4)	8 (13)
Collars	0 (24)	0 (5)	0 (1)	0 (18)
Eye coils	0 (13)	0 (3)		0 (10)
**Any practical constraints**	91	75	83	100
Unavailability of social partners	98	91	100	100
Monitoring of current clinical signs	53	29	0	65
Single housing required imminently	34	11	33	45
Concern over negative consequences (excessive risk)	30	40	0	29
Housing or space limitations	29	11	0	41
Time or staff constraints	25	11	0	33
Information required from research investigator	21	13	0	26
Cost	3	0	0	5
Belief that it does not benefit well-being	0	0	0	0

Note: Blank cells indicates that this constraint is not relevant because the technique or equipment was not reported for this region. Number of facilities responding to each constraint are provided in parentheses.

**Table 6:** Social housing for indoor-housed species in the US over time (percent of facilities). Changes over 5% are indicated via background color. Blue backgrounds denote increases compared to the prior time point, yellow denotes decreases, and white denotes no change.

	Percent of Facilities		
Species	2003	2014	2020
Rhesus macaque	48	50	50
Long-tailed macaque	38	77	77
Pig-tailed macaque	15	42	69
Japanese macaque		48	86
Baboons	7	21	61
Mangabeys	26	19	75
African green/vervet monkey	0	59	62
Common squirrel monkey	33	69	99
Capuchins	86	100	74
Titi monkeys	100	99	98
Common marmoset	78	87	78
Owl monkeys	35	90	88

Note: The blank cell for Japanese macaques in 2003 indicates that no facility reported on this species.

**Table 7:** Constraints to the use of social housing in the US, over time (percent of facilities). Changes over 5% are indicated via background color. Blue backgrounds denote increases compared to the prior time point, yellow denotes decreases, and white denotes no change. The number of facilities responding regarding specific scientific exemptions are provided in parentheses because responses presented here are drawn only from facilities reporting the use of the devices.

Constraints	Percent of Facilities		
	2003	2014	2020
**Clinical exemptions**		51	37
**Behavioral/welfare exemptions**		49	73
**Scientific exemptions**	77	72	83
Tethers		85 (13)	62 (13)
Jackets		57 (21)	0 (13)
Eye coils		45 (11)	0 (10)
Cranial implants		31 (16)	8 (13)
Collars		24 (33)	0 (18)
Implants		21 (19)	6 (16)
**Any practical constraints**	91	95	100
No partner	73	87	100
Time or staffing constraints	32	36	33
Housing or space limitations	41	28	41
Concern over negative consequences		15	29
Single housing required imminently		15	45
Information required from research investigator		8	26
Cost	14	5	5
Belief that it does not benefit well-being		0	0

Note: Blank cells indicates that this constraint is not relevant because the technique or equipment was not queried in the 2003 survey.

**Table 8:** Techniques associated with social housing in the US, over time (percent of facilities). Changes over 5% are indicated via background color. Blue backgrounds denote increases compared to the prior time point, yellow denotes decreases, and white denotes no change.

Technique	2014	2020
Protected contact prior to full contact	96	79
Initial phase of visual access	85	82
Observation of affiliation required to deem introduction successful	48	54
Introduction in neutral cages	44	61
Introduction in enclosures larger than primary housing	41	21
Medication	22	14
Canine blunting	19	18

**Table 9:** USDA minimum enclosure size for NHPs (Animal Welfare Act Section 3.80).

Group	Weight (kg)	Description	Floor Area (m^2^) per Animal	Minimum Height (m^2^) per Animal
1	Under 1	Marmosets, tamarins, and infants (less than 6 months of age) of various species	0.15	0.51
2	1–3	Capuchins, squirrel monkeys and similar size species, and juveniles (6 months to 3 years of age) of various species	0.28	0.76
3	3–10	Macaques and African species	0.40	0.76
4	10–15	Male macaques and large African species	0.56	0.81
5	15–25	Baboons and nonbrachiating species larger than 15 kg	0.74	0.91
6	Over 25	Great apes over 25 kg and brachiating species	2.33	2.13

**Table 10:** European Standards (EU Directive 2010/63/EU, ETS 123 and UK code of practice).

	Minimum Floor Area (m^2^)	Minimum Enclosure Height (m)	Minimum Enclosure Volume (m^3^)	Minimum Volume (m^3^) per Animal
**Macaques and vervets**				
Age < 3 years	2.0	1.8	3.6	1.0
Age from 3 years	2.0	1.8	3.6	1.8
Animals held for breeding		2.0		3.5
**Baboons**				
Age < 4 years	4.0	1.8	7.2	3.0
Age from 4 years	7.0	1.8	12.6	6.0
Animals held for breeding		2.0		3.5
	**Minimum floor area for 1 or 2 animals plus offspring up to 5 months (m^2^)**	**Minimum enclosure height** **(m)**	**Minimum volume per additional animal over 5 months (m^3^)**	
**Marmosets**	0.5	0.2	0.2	
**Tamarins**	1.5	0.2	0.2	
	**Minimum floor area for 1 or 2 animals (m^2^)**	**Minimum enclosure height** **(m)**	**Minimum volume per additional animal over 6 months (m^3^)**	
**Squirrel monkeys**	2.0	1.8	0.5	

**Table 11:** Exercise enclosure schedule, by region (percent of facilities).

Schedule	Percent of Facilities			
	All	EU	UK	US
Daily	41	0	67	41
Several times per week	23	100	33	12
Once a week to once a month	18	0	0	24
Less often than once a month	18	0	0	24

**Table 12:** Constraints on the use of exercise enclosures by region (mean percentage of facilities).

Constraint	All	EU	UK	US
**Scientific exemptions**	5	0	0	7
**Any practical constraints**	70	55	33	85
Space limitations	64	55	33	74
Cost	47	45	0	59
Time or staff constraints	32	0	0	52
Concern over negative consequences	7	0	0	11
Belief that it does not benefit well-being	0	0	0	0

**Table 13:** Schedule of use of exercise enclosures in the US, over time (percent of facilities). Changes over 5% are indicated via background color. Blue backgrounds denote increases compared to the prior time point, yellow denotes decreases, and white denotes no change.

Schedule	2014	2020
Daily	14	46
Several times per week	14	8
Once a week to once a month	38	31
Less often than once a month	29	15

**Table 14:** Constraints on the use of exercise enclosures in the US over time (percent of facilities). Changes over 5% are indicated via background color. Blue backgrounds would denote increases compared to the prior time point, yellow denotes decreases, and white denotes no change.

Constraint	Percent of Facilities	
	2014	2022
**Scientific exemptions**	12	7
**Any practical constraints**	94	85
Space limitations	81	74
Time or staff constraints	62	52
Cost	58	59
Concern over negative consequences	8	11
Belief that it does not benefit well-being	8	0

**Table 15:** Constraints on destructibles, by region (percent of facilities).

Constraint	All	EU	UK	US
**Scientific exemptions**	9	8	0	11
**Any practical constraints**	52	42	0	68
Plumbing or drainage concerns	48	33	0	64
Time or staffing constraints	33	33	0	39
Other impacts on sanitation	26	17	0	36
Cost	15	8	0	21
Concern about potential negative consequences to animals (e.g., injury, stress)	9	0	0	14
Esthetic concerns	4	0	0	7
Belief that destructibles do not benefit well-being	0	0	0	0

**Table 16:** Constraints on the use of destructibles in the US, over time (percent of facilities). Changes over 5% are indicated via background color. Blue backgrounds denote increases compared to the prior time point, yellow denotes decreases, and white denotes no change.

Constraint	Percent of Facilities	
	2014	2020
**Scientific exemptions**	5	11
**Any practical constraints**	80	68
Drainage/plumbing	63	64
Time or staff constraints	46	39
Other impacts on sanitation	41	36
Cost	22	21
Concern about potential negative consequences to animals (e.g., injury, stress)	19	14
Esthetic concerns	7	7
Belief that it does not benefit well-being	0	0

**Table 17:** Constraints on the implementation of sensory enrichment, by region (percent of facilities).

Constraint	All	EU	UK	US
**Scientific exemptions**	0	0	0	0
**Any practical constraints**	51	27	33	65
Time or staff constraints	33	9	17	46
Cost	28	27	0	35
Belief that it does not benefit well-being	12	9	17	12
Concern about potential negative consequences to animals (e.g., injury, stress)	7	9	0	8

**Table 18:** Constraints on the use of sensory enrichment in the US, over time (percent of facilities). Changes over 5% are indicated via background color. Blue backgrounds denote increases compared to the prior time point, yellow denotes decreases, and white denotes no change.

Constraint	2014	2020
**Scientific exemptions**	15	0
**Any practical constraints**	46	65
Time or staff constraints	22	46
Cost	15	35
Concern about potential negative consequences to animals (e.g., injury, stress)	11	8
Belief that it does not benefit well-being	19	12

**Table 19:** Constraints on the implementation of cognitive enrichment (percent of facilities).

Constraint	All	EU	UK	US
**Scientific exemptions**	7	0	0	12
**Any practical constraints**	71	45	55	100
Time or staff constraints	53	45	17	65
Cost	40	18	0	58
Concern about potential negative consequences to animals (e.g., injury, stress)	2	9	0	0
Belief that it does not benefit well-being	2	9	0	0

## Data Availability

The data underlying the results are not openly available due to the inclusion of potentially identifying information. Although no direct identifiers were collected, some responses could allow indirect identification of institutions (e.g., facility characteristics). Given the sensitive nature of non-human primate use, the authors have decided not to share the full dataset; however, sufficient summary data are provided in the manuscript and Supplementary Materials to support the conclusions and interpret the analysis.

## Supplementary Materials

The following supporting information can be downloaded at https://www.mdpi.com/article/10.3390/ani16010138/s1: The survey questionnaire and supplementary tables are provided in the Supplementary Information. Table S1. Percentage of species housed in varying housing configurations, by species and region. Table S2. Percentage of species housed socially: indoor housed animals only. Table S3. Percentage of species housed socially in cages.

## References

[1] Bloomsmith M.A., Schapiro S.J. (2017). Behavioral management of laboratory primates: Principles and projections. Handbook of Primate Behavioral Management.

[2] Bloomsmith M.A., Perlman J.E., Hutchison E., Sharpless M., Weichbrod R.H., Thompson G.A., Norton J.N. (2018). Behavioral management programs to promote laboratory animal welfare. Management of Animal Care and Use Programs in Research, Education, and Testing.

[3] Capitanio J.P. (2021). Knowledge of biobehavioral organization can facilitate better science: A review of the BioBehavioral Assessment Program at the California National Primate Research Center. Animals.

[4] Coleman K., Bloomsmith M., Crockett C., Weed J., Schapiro S. (2012). Chapter 6—Behavioral Management, Enrichment, and Psychological Well-being of Laboratory Nonhuman Primates.

[5] Baker K.C. (2016). Survey of 2014 behavioral management programs for laboratory primates in the United States. Am. J. Primatol..

[6] Prescott M.J., Clark C., Dowling W.E., Shurtleff A.C. (2021). Opportunities for refinement of non-human primate vaccine studies. Vaccines.

[7] Prescott M.J., Lidster K. (2017). Improving quality of science through better animal welfare: The NC3Rs strategy. Lab Anim..

[8] Prior H., Holbrook M. (2021). Strategies to encourage the adoption of social housing during cardiovascular telemetry recordings in non-rodents. J. Pharmacol. Toxicol. Methods.

[9] NIH National Institutes of Health (2020). Fostering Rigorous Research: Lessons Learned from Nonhuman Primate Models and Charting the Path Forward, February 18–19, 2020.

[10] Palmer S., Oppler S.H., Graham M.L. (2022). Behavioral management as a coping strategy for managing stressors in primates: The influence of temperament and species. Biology.

[11] Graham M.L., Schapiro S.J. (2017). Positive reinforcement training and research. Handbook of Primate Behavioral Management.

[12] Buchanan-Smith H.M., Gamble M.R., Gore M., Hawkins P., Hubrecht R., Hudson S., Jennings M., Keeley J.R., Morris K., Members of the Joint Working Group on Refinement (Primates) (2009). Refinements in husbandry, care and common procedures for non-human primates: Ninth report of the BVAAWF/FRAME/RSPCA/UFAW Joint Working Group on Refinement. Lab. Anim..

[13] McMillan J.L., Bloomsmith M.A., Prescott M.J. (2017). An international survey of approaches to chair restraint of nonhuman primates. Comp. Med..

[14] Russell W.M.S., Burch R.L. (1959). The Principles of Humane Experimental Technique.

[15] NRC (National Research Council) (2011). Guide for the Care and Use of Laboratory Animals.

[16] EU. European Union (2010). Directive 2010/63/EU of the European Parliament and of the Council of 22 September 2010 on the Protection of Animals Used for Scientific Purposes. Off. J. Eur. Union.

[17] EU. Council of Europe (2006). Appendix A of the European Convention for the Protection of Vertebrate Animals Used for Experimental and Other Scientific Purposes (ETS 123). Guidelines for Accommodation and Care of Animals (Article 5 of the Convention). Approved by the Multilateral Consultation. Cons 123 (2006) 3. Strasbourg: Council of Europe. https://www.coe.int/t/e/legal_affairs/legal_co-operation/biological_safety_and_use_of_animals/laboratory_animals/revision%20of%20appendix%20a.asp.

[18] NC3Rs (2017). NC3Rs Guidelines: Non-Human Primate Accommodation, Care and Use.

[19] Baker K.C., Weed J.L., Crockett C.M., Bloomsmith M.A. (2007). Survey of environmental enhancement programs for laboratory primates. Am. J. Primatol..

[20] HO. UK Home Office (2014). Code of Practice for the Housing and Care of Animals Bred, Supplied or Used for Scientific Purposes. https://www.gov.uk/government/publications/code-of-practice-for-the-housing-and-care-of-animals-bred-supplied-or-used-for-scientific-purposes.

[21] USDA (United States Department of Agriculture) (1991). Code of Federal Regulations. Title 9, Part 3, Animal welfare; Standards; Final Rule. Fed. Reg..

[22] Richardson S. (2024). Primate enrichment categories: A literature review of current trends. Anim. Behav. Cogn..

[23] Cinque C., De Marco A., Mairesse J., Giuli C., Sanna A., De Marco L., Zuena A.R., Casolini P., Catalani A., Thierry B. (2017). Relocation stress induces short-term fecal cortisol increase in Tonkean macaques (*Macaca tonkeana*). Primates.

[24] Fernstrom A.L., Sutian W., Royo F., Westlund K., Nilsson T., Carlsson H.E., Paramastri Y., Pamungkas J., Sajuthi D., Schapiro S.J. (2008). Stress in cynomolgus monkeys (*Macaca fascicularis*) subjected to long-distance transport and simulated transport housing conditions. Stress.

[25] Honess P.E., Johnson P.J., Wolfensohn S.E. (2004). A study of behavioural responses of non-human primates to air transport and re-housing. Lab. Anim..

[26] Kagira J.M., Ngotho M., Thuita J.K., Maina N.W., Hau J. (2007). Hematological changes in vervet monkeys (Chlorocebus aethiops) during eight months’ adaptation to captivity. Am. J. Primatol..

[27] Kim C.Y., Han J.S., Suzuki T., Han S.S. (2005). Indirect indicator of transport stress in hematological values in newly acquired cynomolgus monkeys. J. Med. Primatol..

[28] Nehete P.N., Nehete B.P., Patel A.G., Chitta S., Scholtzova H., Williams L.E. (2021). Short-term relocation stress-induced hematological and immunological changes in *Saimiri boliviensis boliviensis*. J. Immunol. Res..

[29] Shelton K.A., Nehete B.P., Chitta S., Williams L.E., Schapiro S.J., Simmons J., Abee C.R., Nehete P.N. (2019). Effects of transportation and relocation on immunologic measures in cynomolgus macaques (*Macaca fascicularis*). J. Am. Assoc. Lab. Anim. Sci..

[30] Watson S.L., McCoy J.G., Stavisky R.C., Greer T.F., Hanbury D. (2005). Cortisol response to relocation stress in Garnett’s bushbaby (*Otolemur garnettii*). Contemp. Top. Lab. Anim. Sci..

[31] Wolfensohn S.E. (1997). Brief review of scientific studies of the welfare implications of transporting primates. Lab. Anim..

[32] Logan L.E., Sayers K. (2024). Pairing Laboratory-housed adult male rhesus macaques (*Macaca mulatta*): Success rates in relation to behavioral response and duration of visual contact. Appl. Anim. Behav. Sci..

[33] MacAllister R.P., Heagerty A., Coleman K. (2020). Behavioral predictors of pairing success in rhesus macaques (*Macaca mulatta*). Am. J. Primatol..

[34] Pomerantz O., Baker K.C., Bellanca R.U., Bloomsmith M.A., Coleman K., Hutchinson E.K., Pierre P.J., Weed J.L., National Primate Research Centers’ Behavioral Management Consortium (2022). Improving transparency—A call to include social housing information in biomedical research articles involving nonhuman primates. Am. J. Primatol..

[35] Pomerantz O., Capitanio J.P. (2021). Temperament predicts the quality of social interactions in captive female rhesus macaques (*Macaca mulatta*). Animals.

[36] Bailey K.L., Young L.A., Long C.E., Remillard C.M., Moss S.E., Meeker T.L., Bloomsmith M.A. (2021). Use of introduction enclosures to integrate multimale cohorts into groups of female rhesus macaques (*Macaca mulatta*). J. Am. Assoc. Lab. Anim. Sci..

[37] Beisner B.A., Remillard C.M., Moss S., Long C.E., Bailey K.L., Young L.A., Meeker T., McCowan B., Bloomsmith M.A. (2021). Factors influencing the success of male introductions into groups of female rhesus macaques: Introduction technique, male characteristics and female behavior. Am. J. Primatol..

[38] Rox A., van Vliet A.H., Sterck E.H.M., Langermans J.A.M., Louwerse A.L. (2019). Factors determining male introduction success and long-term stability in captive rhesus macaques. PLoS ONE.

[39] Kezar S.M., Baker K.C., Russell-Lodrigue K.E., Bohm R.P. (2022). Single-dose diazepam administration improves pairing success of unfamiliar adult male rhesus macaques (*Macaca mulatta*). J. Am. Assoc. Lab. Anim. Sci..

[40] Jorgensen M.J., Lambert K.R., Breaux S.D., Baker K.C., Snively B.M., Weed J.L. (2017). Pair housing of vervets/African green monkeys for biomedical research. Am. J. Primatol..

[41] Baker K.C., Crockett C.M., Lee G.H., Oettinger B.C., Schoof V., Thom J.P. (2012). Pair housing for female longtailed and rhesus macaques in the laboratory: Behavior in protected contact versus full contact. J. Appl. Anim. Welf. Sci..

[42] Pomerantz O., Baker K.C. (2017). Higher levels of submissive behaviors at the onset of the pairing process of rhesus macaques (*Macaca mulatta*) are associated with lower risk of wounding following introduction. Am. J. Primatol..

[43] Doyle L.A., Baker K.C., Cox L.D. (2008). Physiological and behavioral effects of social introduction on adult male rhesus macaques. Am. J. Primatol..

[44] Murti A.M., Wilson C.C., Pemberton A.F., Corey T.M., Dzikiti L.N., Elsworth J.D., Carpenter C.B. (2024). Factors that determine successful social housing of African green monkeys (*Chlorocebus sabaeus*) in same-sex pairs and trios. Vet. Sci..

[45] Coleman K., Timmel G., Prongay K., Baker K.C. (2023). Common husbandry, housing, and animal care practices. Nonhuman Primate Welfare: From History, Science, and Ethics to Practice.

[46] Nederlof R.A., Bruins-van Sonsbeek L.G., Stumpel J.B., Bakker J. (2024). Update on current hormonal and non-hormonal contraceptive options in non-human primates. J. Zool. Bot. Gard..

[47] Wallis J., Valentine B. (2001). Early vs. natural weaning in captive baboons: The effect on timing of postpartum estrus and next conception. Lab. Primate Newsl..

[48] Crast J., Bloomsmith M.A., Remillard C.M., Meeker T. (2021). Contribution of adult sex ratio to trauma and reproductive output in large breeding groups of rhesus macaques (*Macaca mulatta*). Anim. Welf..

[49] Johnson-Delaney C.A. (2008). Nonhuman primate dental care. J. Exot. Pet. Med..

[50] Hannibal D., Beisner B., Cital S., Maness A., Kashyap N., McCowan B., Sammak R. Effect of adult male canine tooth modification on group welfare in rhesus macaques. Proceedings of the 42nd Association of Primate Veterinarians Workshop.

[51] Sammak R., Meier C., Keesler R. Canine tooth pathology following canine crown reduction and canine blunting in rhesus macaques. Proceedings of the 43rd Association of Primate Veterinarians Workshop.

[52] APV Association of Primate Veterinarians (2022). Association of Primate Veterinarians Guidelines for Wound Management of Nonhuman Primates. J. Am. Assoc. Lab. Anim. Sci..

[53] AVMA American Veterinary Medical Association (2012). Removal or Reduction of Teeth in Nonhuman Primates and Carnivores. https://www.avma.org/javma-news/2013-01-15/single-policy-teeth-reduction-and-removal-adopted.

[54] Hopper L.M., Allen J.V., Huynh V., Painter M.C., Izzi J., Hutchinson E.K. (2024). The use of guanfacine to mediate anxiety-related reactivity and reduce associated agonistic behavior in two pigtail macaques (*Macaca nemestrina*). Comp. Med..

[55] Myers A., Baker K., Kubisch M., Russell-Lodrigue K., Bohm R. (2025). Evaluating outcomes of diazepam administration in gradual steps introductions of adult male and female rhesus macaques (*Macaca mulatta*). J. Am. Assoc. Lab. Anim. Sci..

[56] Berg M.R., Heagerty A., Coleman K. (2019). Oxytocin and pair compatibility in adult male rhesus macaques (*Macaca mulatta*). Am. J. Primatol..

[57] Snowdon C.T., Pieper B.A., Boe C.Y., Cronin K.A., Kurian A.V., Ziegler T.E. (2010). Variation in oxytocin is related to variation in affiliative behavior in monogamous, pairbonded tamarins. Horm. Behav..

[58] Benitez M.E., Sosnowski M.J., Tomeo O.B., Brosnan S.F. (2018). Urinary oxytocin in capuchin monkeys: Validation and the influence of social behavior. Am. J. Primatol..

[59] Smith A.S., Agmo A., Birnie A.K., French J.A. (2010). Manipulation of the oxytocin system alters social behavior and attraction in pair-bonding primates, *Callithrix penicillata*. Horm. Behav..

[60] Zablocki-Thomas P., Lau A., Witczak L., Dufek M., Wright A., Savidge L., Paulus J., Baxter A., Karaskiewicz C., Seelke A.M.H. (2023). Intranasal oxytocin does not change partner preference in female titi monkeys (*Plecturocebus cupreus*), but intranasal vasopressin decreases it. J. Neuroendocrinol..

[61] Coppeto D.J., Martin J.S., Ringen E.J., Palmieri V., Young L.J., Jaeggi A.V. (2024). Peptides and primate personality: Central and peripheral oxytocin and vasopressin levels and social behavior in two baboon species (*Papio hamadryas* and *Papio anubis*). Peptides.

[62] Jiang Y., Platt M.L. (2018). Oxytocin and vasopressin flatten dominance hierarchy and enhance behavioral synchrony in part via anterior cingulate cortex. Sci. Rep..

[63] Passini E., Adedeji Adeyemi O., Allen R., Baldrick P., Basak J., Britton L., Cotton P., Harding J., Holmes T., Ljumanovic N. (2025). Applying the 3Rs to urinalysis assessments in toxicity studies: Refining procedures and adopting a case-by-case approach. Comp. Clin. Pathol..

[64] McKinley J., Buchanan-Smith H.M., Bassett L., Morris K. (2003). Training common marmosets (*Callithrix jacchus*) to cooperate during routine laboratory procedures: Ease of training and time investment. J. Appl. Anim. Welf. Sci..

[65] Smith T.E., McCallister J.M., Gordon S.J., Whittikar M. (2004). Quantitative data on training new world primates to urinate. Am. J. Primatol..

[66] Stow R., Kendrick J., Ibbotson N., Adjin-Tettey G., Murphy B., Trojca R., Miller J., Konradsen G., Ovlisen K., Helleberg H. (2021). A new group housing approach for non-human primate metabolism studies. J. Pharmacol. Toxicol. Methods.

[67] Castell N., Guerrero-Martin S.M., Rubin L.H., Shirk E.N., Brockhurst J.K., Lyons C.E., Najarro K.M., Queen S.E., Carlson B.W., Adams R.J. (2022). Effect of single housing on innate immune activation in immunodeficiency virus-infected pigtail macaques (*Macaca nemestrina*) as a model of psychosocial stress in acute HIV infection. Psychosom. Med..

[68] Guerrero-Martin S.M., Rubin L.H., McGee K.M., Shirk E.N., Queen S.E., Li M., Bullock B., Carlson B.W., Adams R.J., Gama L. (2020). Social stress alters immune response and results in higher viral load during acute SIV infection in a pigtailed macaque model of HIV. bioRxiv.

[69] Guerrero-Martin S.M., Rubin L.H., McGee K.M., Shirk E.N., Queen S.E., Li M., Bullock B., Carlson B.W., Adams R.J., Gama L. (2021). Psychosocial stress alters the immune response and results in higher viral load during acute simian immunodeficiency virus infection in a pigtailed macaque model of human immunodeficiency virus. J. Infect. Dis..

[70] Prescott M.J., Brown V.J., Flecknell P.A., Gaffan D., Garrod K., Lemon R.N., Parker A.J., Ryder K., Schultz W., Scott L. (2010). Refinement of the use of food and fluid control as motivational tools for macaques used in behavioural neuroscience research: Report of a Working Group of the NC3Rs. J. Neurosci. Methods.

[71] Paterson E.A., O’Malley C.I., Abney D.M., Archibald W.J., Turner P.V. (2024). Development of a novel primate welfare assessment tool for research macaques. Anim. Welf..

[72] Nunamaker E.A., Malinowski C.M., Goodroe A.E., Guerriero K.A., Burns M.A. (2023). Anesthesia and analgesia in nonhuman primates. Anesthesia and Analgesia in Laboratory Animals.

[73] Coelho A.M., Carey K.D. (1990). A social tethering system for nonhuman primates used in laboratory research. Lab. Anim. Sci..

[74] Coelho A.M., Carey K.D., Shade R.E. (1991). Assessing the effects of social environment on blood pressure and heart rates of baboons. Am. J. Primatol..

[75] McNamee G.A., Wannemacher R.W., Dinterman R.E., Rozmiarek H., Montrey R.D. (1984). A surgical procedure and tethering system for chronic blood sampling, infusion, and temperature monitoring in caged nonhuman primates. Lab. Anim. Sci..

[76] Crockett C.M., Bowers C.L., Sackett G.P., Bowden D.M. (1993). Urinary cortisol responses of longtailed macaques to five cage sizes, tethering, sedation, and room change. Am. J. Primatol..

[77] Martin L.D., Shelton J., Houser L.A., MacAllister R., Coleman K. (2024). Refinements in clinical and behavioral management for macaques on infectious disease protocols. Vet. Sci..

[78] Kaiser R.A., Tichenor S.D., Regalia D.E., York K., Holzgrefe H.H. (2015). Telemetric assessment of social and single housing: Evaluation of electrocardiographic intervals in jacketed cynomolgus monkeys. J. Pharmacol. Toxicol. Methods.

[79] Roberts S.J., Platt M.L. (2005). Effects of isosexual pair-housing on biomedical implants and study participation in male macaques. Contemp. Top. Lab. Anim. Sci..

[80] Massey D.A. (2024). Delaying the Weaning of Captive Rhesus Macaques (*Macaca mulatta*) Destined for Use in Neuroscience Research: An Assessment of the Costs and Benefits. Doctoral Dissertation.

[81] Prescott M.J., Nixon M.E., Farningham D.A., Naiken S., Griffiths M.A. (2012). Laboratory macaques: When to wean?. Appl. Anim. Behav. Sci..

[82] Rox A., Waasdorp S., Sterck E.H.M., Langermans J.A.M., Louwerse A.L. (2022). Multigenerational social housing and group-rearing enhance female reproductive success in captive rhesus macaques (*Macaca mulatta*). Biology.

[83] Conti G., Hansman C., Heckman J.J., Novak M.F., Ruggiero A., Suomi S.J. (2012). Primate evidence on the late health effects of early-life adversity. Proc. Natl. Acad. Sci. USA.

[84] Lutz C., Well A., Novak M. (2003). Stereotypic and self-injurious behavior in rhesus macaques: A survey and retrospective analysis of environment and early experience. Am. J. Primatol..

[85] Rommeck I., Gottlieb D.H., Strand S.C., McCowan B. (2009). The effects of four nursery rearing strategies on infant behavioral development in rhesus macaques (*Macaca mulatta*). J. Am. Assoc. Lab. Anim. Sci..

[86] Wood E.K., Kruger R., Day J.P., Day S.M., Hunter J.N., Neville L., Lindell S.G., Barr C.S., Schwandt M.L., Goldman D. (2022). A nonhuman primate model of human non-suicidal self-injury: Serotonin-transporter genotype-mediated typologies. Neuropsychopharmacology.

[87] Dettmer A.M., Chusyd D.E. (2023). Early life adversities and lifelong health outcomes: A review of the literature on large, social, long-lived nonhuman mammals. Neurosci. Biobehav. Rev..

[88] Strand S.C., Novak M.A. (2005). Examination of behavior in differently reared monkeys housed together. Am. J. Primatol..

[89] Higley J.D., Linnoila M., Suomi S.J., Hersen M., Ammerman R.T., Sisson L. (1994). Ethological contributions: Experiential and genetic contributions to the expression and inhibition of aggression in primates. Handbook of Aggressive and Destructive Behavior in Psychiatric Patients.

[90] Novak M.F., Sackett G.P. (1997). Pair-rearing infant monkeys (*Macaca nemestrina*) using a “rotating-peer” strategy. Am. J. Primatol..

[91] Ruppenthal G.C., Walker C.G., Sackett G.P. (1991). Rearing infant monkeys (*Macaca nemestrina*) in pairs produces deficient social development compared with rearing in single cages. Am. J. Primatol..

[92] Sproul Bassett A.M., Wood E.K., Lindell S.G., Schwandt M.L., Barr C.S., Suomi S.J., Higley J.D. (2020). Intergenerational effects of mother’s early rearing experience on offspring treatment and socioemotional development. Dev. Psychobiol..

[93] Capitanio J.P., Mason W.A., Mendoza S.P., DelRosso L., Robers J.A., Sackett G.P., Elizas K. (2006). Nursery rearing and biobehavioral organization. Nursery Rearing of Nonhuman Primates in the 21st Century.

[94] Coe C.L. (1993). Psychosocial factors and immunity in nonhuman primates: A review. Psychosom. Med..

[95] Gottlieb D.H., Capitanio J.P., McCowan B. (2013). Risk factors for stereotypic behavior and self-biting in rhesus macaques (*Macaca mulatta*): Animal’s history, current environment, and personality. Am. J. Primatol..

[96] Tromp D.P.M., Fox A.S., Riedel M.K., Oler J.A., Zhou X., Roseboom P.H., Alexander A.L., Kalin N.H. (2024). Early life adversity in primates: Behavioral, endocrine, and neural effects. Psychoneuroendocrinology.

[97] Ash H., Smith T.E., Buchanan-Smith H.M. (2021). The long-term impact of infant rearing background on the behavioural and physiological stress response of adult common marmosets (*Callithrix jacchus*). affective state of adult common marmosets (*Callithrix jacchus*). Appl. Anim. Behav. Sci..

[98] Brent L., Bode A. (2006). Baboon nursery rearing practices and comparisons between nursery-reared and mother-reared individuals. Nursery Rearing of Nonhuman Primates in the 21st Century.

[99] Mulholland M.M., Williams L.E., Abee C.R. (2020). Rearing condition may alter neonatal development of captive Bolivian squirrel monkeys (*Saimiri boliviensis boliviensis*). Dev. Psychobiol..

[100] Neal S.J., Schapiro S.J., Lambeth S.P., Magden E.R. (2024). Nursery- vs. mother-reared baboons: Reproductive success and health parameters. Vet. Sci..

[101] Buchanan-Smith H., Prescott M., Cross N. (2004). What factors should determine cage sizes for primates in the laboratory?. Anim. Welf..

[102] Prescott M.J., Buchanan-Smith H.M. (2004). Cage sizes for tamarins in the laboratory. Anim. Welf..

[103] Wolfensohn S.E., Honess P. (2005). Handbook of Primate Husbandry and Welfare.

[104] Beisner B.A., Hannibal D.L., Vandeleest J.J., McCowan B. (2023). Sociality, health, and welfare in nonhuman primates. Nonhuman Primate Welfare: From History, Science, and Ethics to Practice.

[105] Buchanan-Smith H.M., Shand C., Morris K. (2002). Cage use and feeding height preferences of captive common marmosets (*Callithrix j. jacchus*) in two-tier cages. J. Appl. Anim. Welf. Sci..

[106] Clarence W.M., Scott J.P., Dorris M.C., Pare M. (2006). Use of enclosures with functional vertical space by captive rhesus monkeys (*Macaca mulatta*) involved in biomedical research. J. Am. Assoc. Lab. Anim. Sci..

[107] Kitchen A.M., Martin A.A. (1996). The effects of cage size and complexity on the behaviour of captive common marmosets, *Callithrix jacchus* jacchus. Lab. Anim..

[108] MacLean E.L., Prior S.R., Platt M.L., Brannon E.M. (2009). Primate location preference in a double-tier cage: The effects of illumination and cage height. J. Appl. Anim. Welf. Sci..

[109] Griffis C.M., Martin A.L., Perlman J.E., Bloomsmith M.A. (2013). Play caging benefits the behavior of singly housed laboratory rhesus macaques (*Macaca mulatta*). J. Am. Assoc. Lab. Anim. Sci..

[110] Wolfensohn S. (2004). Social housing of large primates: Methodology for refinement of husbandry and management. Altern. Lab. Anim..

[111] Anderson J., Chamove A. (1984). Allowing Captive Primates to Forage, Standards in Laboratory Animal Management.

[112] Blois-Heulin C., Jubin R. (2004). Influence of the presence of seeds and litter on the behaviour of captive red-capped mangabeys Cercocebus torquatus torquatus. Appl. Anim. Behav. Sci..

[113] Boccia M., Hijazi A. (1998). A foraging task reduces agonistic and stereotypic behaviors in pigtail macaques. Lab. Primate Newsl..

[114] Chamove A., Anderson J., Morgan-Jones S., Jones S. (1982). Deep woodchip litter: Hygiene, feeding, and behavioral enhancement in 8 primate species. Int. J. Study Anim. Probl..

[115] Doane C.J., Andrews K., Schaefer L.J., Morelli N., McAllister S., Coleman K. (2013). Dry bedding provides cost-effective enrichment for group-housed rhesus macaques (*Macaca mulatta*). J. Am. Assoc. Lab. Anim. Sci..

[116] Janavaris M., Bader L., Hansen J.J., Bodvarsdottir T.B., Coleman K., Kievit P. (2022). Bedding as an enrichment strategy in group-housed Mauritian cynomolgus macaques (*Macaca fascicularis*). J. Am. Assoc. Lab. Anim. Sci..

[117] Bennett A.J., Corcoran C.A., Hardy V.A., Miller L.R., Pierre P.J. (2010). Multidimensional cost-benefit analysis to guide evidence-based environmental enrichment: Providing bedding and foraging substrate to pen-housed monkeys. J. Am. Assoc. Lab. Anim. Sci..

[118] Meade T.M., Hutchinson E., Krall C., Watson J. (2014). Use of an aquarium as a novel enrichment item for singly housed rhesus macaques (*Macaca mulatta*). J. Am. Assoc. Lab. Anim. Sci..

[119] Schapiro S.J., Bloomsmith M.A. (1995). Behavioral effects of enrichment on singly-housed, yearling rhesus monkeys: An analysis including three enrichment conditions and a control group. Am. J. Primatol..

[120] Schapiro S.J., Porter L.M., Suarez S.A., Bloomsmith M.A. (1995). The behavior of singly-caged, yearling rhesus monkeys is affected by the environment outside of the cage. Appl. Anim. Behav. Sci..

[121] Hanbury D.B., Fontenot M.B., Highfill L.E., Bingham W., Bunch D., Watson S.L. (2009). Efficacy of auditory enrichment in a prosimian primate (*Otolemur garnettii*). Lab Anim..

[122] Brent L., Weaver D. (1996). The physiological and behavioral effects of radio music on singly housed baboons. J. Med. Primatol..

[123] dos Santos A.M., da Silva M.B., Prado I.S., de Antonio E.S., Dourado A.T.T.S., Alves B.S., Nishiyama P.B., Tomazi L., Fraga R.E. (2022). Evaluation of sound enrichment in the behavior of *Sapajus xanthosternos* (Wied-Neuwied, 1826) (Primates: Cebidae) in Captivity. Primate Conserv..

[124] Line S.W., Clarke A.S., Markowitz H., Ellman G. (1990). Responses of female rhesus macaques to an environmental enrichment apparatus. Lab. Anim..

[125] Ogura T., Matsuzawa T. (2012). Video preference assessment and behavioral management of single-caged Japanese macaques (*Macaca fuscata*) by movie presentation. J. Appl. Anim. Welf. Sci..

[126] Platt D.M., Novak M.A. (1997). Video stimulation as enrichment for captive rhesus monkeys (*Macaca mulatta*). Appl. Anim. Behav. Sci..

[127] Prescott M.J. (2006). Primate Sensory Capabilities and Communication Signals: Implications for Care and Use in the Laboratory.

[128] Baxter A., Lau A.R., Savidge L.E., Bales K.L. (2023). Initial compatibility during a “Speed-Dating” test predicts postpairing affiliation in titi monkeys (*Plecturocebus cupreus*). Am. J. Primatol..

[129] Stull C., Heagerty A., Coleman K. (2022). Video conference technology as a tool for pair introduction in rhesus macaques. Animals.

[130] de la Chica A.G., Fernandez-Duque E., Williams L. (2021). Behavioral Biology of Owl Monkeys. Behavioral Biology of Laboratory Animals.

[131] Tardif S., Bales K., Williams L., Moeller E.L., Abbott D., Schultz-Darken N., Mendoza S., Mason W., Bourgeois S., Ruiz J. (2006). Preparing New World monkeys for laboratory research. ILAR J..

[132] Buchanan-Smith H. (2010). Environmental enrichment for primates in laboratories. Adv. Sci. Res..

[133] Butler J.L., Kennerley S.W. (2019). Mymou: A low-cost, wireless touchscreen system for automated training of nonhuman primates. Behav. Res. Methods.

[134] Calapai A., Berger M., Niessing M., Heisig K., Brockhausen R., Treue S., Gail A. (2017). A cage-based training, cognitive testing and enrichment system optimized for rhesus macaques in neuroscience research. Behav. Res. Methods.

[135] Womelsdorf T., Thomas C., Neumann A., Watson M.R., Banaie Boroujeni K., Hassani S.A., Parker J., Hoffman K.L. (2021). A kiosk station for the assessment of multiple cognitive domains and cognitive enrichment of monkeys. Front. Behav. Neurosci..

[136] Calapai A., Pfefferle D., Cassidy L.C., Nazari A., Yurt P., Brockhausen R.R., Treue S. (2023). A touchscreen-based, multiple-choice approach to cognitive enrichment of captive rhesus macaques (*Macaca mulatta*). Animals.

[137] Fagot J., Gullstrand J., Kemp C., Defilles C., Mekaouche M. (2014). Effects of freely accessible computerized test systems on the spontaneous behaviors and stress level of Guinea baboons (*Papio papio*). Am. J. Primatol..

[138] Lincoln H., Andrews M.W., Rosenblum L.A. (1995). Pigtail macaque performance on a challenging joystick task has important implications for enrichment and anxiety within a captive environment. Lab. Anim. Sci..

[139] Washburn D.A., Rumbaugh D.M. (1992). Investigations of rhesus monkey video-task performance: Evidence for enrichment. Contemp. Top. Lab. Anim. Sci..

[140] Griggs D.J., Bloch J., Chavan S., Coubrough K.M., Conley W., Morrisroe K., Yazdan-Shahmorad A. (2021). Autonomous cage-side system for remote training of non-human primates. J. Neurosci. Methods.

[141] Prescott M.J., Leach M.C., Truelove M.A. (2023). Harmonisation of welfare indicators for macaques and marmosets used or bred for research. F1000Research.

[142] Truelove M.A., Martin J.E., Langford F.M., Leach M.C. (2020). The identification of effective welfare indicators for laboratory-housed macaques using a Delphi consultation process. Sci. Rep..

