# Supplementary material for: Evaluating Behavioral Management Practices for Laboratory Nonhuman Primates: An International Survey
Source: Animals (Basel). 2026 Jan 3;16(1):138. doi: 10.3390/ani16010138 (PMC12784784; doi:10.3390/ani16010138)
Supplement: Supplementary file 1 [file animals-16-00138-s001.zip › Behavioural management survey - supplementary tables proofed.pdf]

Table S1. Percentage of species housed in varying housing configurations, by species and region.

	All regions	EU	UK	US
**Single**	**15**	**5**	**1**	**16**
African green monkey	27	0		27
Common marmoset	20	7		21
Rhesus macaque	19	14	12	19
Long-tailed macaque	8	5	0	9
Capuchins	7	0		22
Baboons	3	1		3
Common squirrel monkey	1	0		1
Pig-tailed macaque				18
Mangabeys				10
Owl monkeys				9
Titi monkeys				2
Japanese macaque				1
**Protected contact**	**1**	**1**	**3**	**1**
Rhesus macaque	1	<1	27	1
Common marmoset	<1	0		<1
Long-tailed macaque	<1	1	0	<1
Baboons	<1	0		<1
Common squirrel monkey	<1	0		<1
Capuchins	0	0		0
Pig-tailed macaque				4
Mangabeys				3
Owl monkeys				1
Titi monkeys				0
Japanese macaque				0
African green monkey	0	0		0
**Intermittent pair**	**3**	**1**	**0**	**3**
Long-tailed macaque	12	0	0	14
Baboons	1	0		1
Rhesus macaque	<1	5	0	<1
Pig-tailed macaque	<1			<1
African green monkey	0	0		0
Common squirrel monkey	0	0		0
Capuchins	0	0		0
Common marmoset	0	0		0
Owl monkey				1
Japanese macaque				0
Mangabeys				0
Titi monkeys				0

**Continuous pair**	**27**	**22**	**9**	**28**
Long-tailed macaque	51	28	<1	58
African green monkey	35	13		35
Common marmoset	30	20		35
Rhesus macaque	16	19	34	16
Common squirrel monkey	10	0		11
Capuchins	9	2		22
Baboons	3	7		3
Titi monkeys				47
Owl monkeys				45
Pig-tailed macaque				23
Mangabeys				1
Japanese macaque				1
**Indoor group housing**	**11**	**32**	**86**	**9**
Common squirrel monkey	81	24		87
Common marmoset	41	74		28
Long-tailed macaque	23	34	99	18
Capuchins	13	0		39
African green monkey	8	0		8
Baboons	3	0		4
Rhesus macaque	2	17	29	2
Titi monkeys				51
Owl monkeys				44
Mangabeys				39
Pig-tailed macaque				25
Japanese macaque				4
**Indoor group housing with access to the out of doors**	**9**	**34**	**0**	**8**
Capuchins	44	57		17
African green monkey	31	87		30
Baboons	30	92		18
Common squirrel monkey	8	76		0
Rhesus macaque	8	40	0	8
Long-tailed macaque	5	27	0	2
Common marmoset	0	0		0
Pig-tailed macaque				30
Mangabeys				29
Japanese macaque				23
Titi monkeys				0
Owl monkeys				0
**Outdoor group housing**	**33**	**6**	**0**	**36**
Baboons	59	0		70
Rhesus macaque	54	5	0	55

Capuchins	27	40		0
Long-tailed macaque	1	5	0	0
African green/vervet monkey	0	0		0
Common squirrel monkey	0	0		0
Common marmoset	0	0		0
Japanese macaque				71
Mangabeys				18
Pig-tailed macaque				0
Titi monkeys				0
Owl monkeys				0

Note: Cells in the ‘All regions’ column are blank where there are data from only one region. Other cells are blank for regions in which a species is not housed.

Table S2. Percentage of species housed socially: indoor housed animals only.

	All regions	EU	UK*	US
**All species**	67	89	96*	65
Common squirrel monkey	99	100		99
Common marmoset	83	93		78
Long-tailed macaque	79	91	100	77
Capuchins	75	100		74
Baboons	64	86		61
African green/vervet monkey	62	100		62
Rhesus macaque	51	65	62	50
Titi monkeys				98
Owl monkeys				88
Japanese macaque				86
Mangabeys				75
Pig-tailed macaque				69

* UK values are identical to Table S1 because all animals reported were housed indoors.

Note: Cells in the ‘All regions’ column are blank where species were reported only one region. Other cells are blank for regions in which a species is not housed.

Table S3. Percentage of species housed socially in cages.

	**All regions**	**EU**	**UK**	**US**
**All**	**59**	**76**	**69**	**58**
Long-tailed macaque	72	82	100	72
Common marmoset	71	75		70
African green/vervet monkey	57	100		57
Capuchins	55	100		50
Baboons	47	86		39
Rhesus macaque	47	49	47	47
Titi monkeys				96
Common squirrel monkey				89
Owl monkeys				79
Japanese macaque				63
Pig-tailed macaque				51
Mangabeys				8

Note: Cells in the ‘All regions’ column are blank where there are data from only one region. Other cells are blank for regions in which a species is not housed.
