# Supplementary material for: Evaluating Behavioral Management Practices for Laboratory Nonhuman Primates: An International Survey
Source: Animals (Basel). 2026 Jan 3;16(1):138. doi: 10.3390/ani16010138 (PMC12784784; doi:10.3390/ani16010138)
Supplement: Supplementary file 1 [file animals-16-00138-s001.zip › Supplementary info - survey questions proofed.pdf]

Welcome

You are invited to take part in a survey to collect information on behavioral management of nonhuman primates (NHPs). The survey is a collaboration between the Tulane National Primate Research Center (TNPRC) in the USA and National Centre for the Replacement, Refinement and Reduction of Animals in Research (NC3Rs) in the UK. We very much hope you will participate.

The aims are to:

- **Gather information on behavioral management practices currently in use.**
- **Understand changes in behavioral management practices over time.**
- **Generate an evidence base to inform and support good practice.**
- **Disseminate the findings and recommendations via publication and conference presentations.**

The survey consists of 77 questions and we estimate it will take 50 minutes to complete. A PDF of the questions is here, if you would like to prepare your answers offline first (a few questions may require reviewing records).

Please limit responses to one representative from your laboratory (or laboratory members can work on as a group), as this will decrease any chance of repetition.

The survey will close on 20 December 2019 at 24:00 GMT.

All data collected in this survey will be anonymous, treated in strict confidence and held securely by the TNPRC and NC3Rs. The NC3Rs data management plan is available upon request (enquiries@nc3rs.org.uk)

If you have any questions or concerns about the survey, please contact Dr Kate Baker (kbaker1@tulane.edu) or Dr Mark Prescott (mark.prescott@nc3rs.org.uk).

Thank you in advance for your participation.

Consent
You must be at least 18 years old to participate. You are free to withdraw at any time and without giving a reason. There are no known benefits or risks for you in this survey.

Please indicate your consent to take part in the survey by reading the following statement and selecting the appropriate option below. By selecting 'yes', you are confirming that:

- **You are at least 18 years of age.**
- **You understand the purpose of the survey and that you can ask questions about it at any time.**
- **You understand you are free to withdraw at any time.**
- **You understand that your name will not appear in any published document relating to this study as all information collected is anonymous.**

By continuing and submitting a response to the survey, you have read and understood the

above and have given informed consent.

1. Do you consent to participate?

◯ Yes

◯ No

Background information

Behavioral management: a holistic method for improving animal care and welfare that integrates species-appropriate facility design, husbandry, environmental enrichment, animal training, and behavioral monitoring and assessment.

2. Facility category?

- ○ United States National Primate Research Center
- ○ University-affiliated primate facility
- ○ Private laboratory (e.g. industry, contract research)
- ○ Breeding facility only
- ○ Government-funded laboratory (e.g., National Institutes of Health)

Other (please specify):

[ ]

3. In which country do you work? (free text)

[ ]

4. Who is responsible for the oversight of your behavioral management program for NHPs?

- ○ Veterinarian
- ○ Animal technician or welfare officer (e.g. NACWO)
- ○ Other dedicated individual (e.g. behavioral management or enrichment coordinator)
- ○ Multiple individuals working independently (i.e., separate investigators' labs, no facility-wide umbrella of oversight)
- ○ Dedicated committee; include number of individuals in group
- ○ Other (please specify):

[ ]

5. What is the highest degree or qualification held by the person responsible for the oversight of your behavioral management program?

- ○ High school degree / GCSEs (A*-C)
- ○ A levels / HNC / IB / EB
- ○ Associate's degree / Foundation degree
- ○ Bachelor's degree
- ○ Master's degree
- ○ PhD
- ○ DVM
- ○ Other (please specify):

[ ]

6. Does your facility employee dedicated staff who perform only behavioral management-related duties?

- ○ Yes
- ○ No

7. If so, what are the highest degrees held by individual behavioral management employees? (tick all that apply)

- ☐ High school degree / GCSEs (A*-C)
- ☐ A levels / HNC / IB / EB
- ☐ Associate's degree / Foundation degree
- ☐ Bachelor's degree
- ☐ Other (please specify):
- ☐ Master’s degree
- ☐ PhD
- ☐ DVM

Social housing

Social housing: animals kept with one or more compatible conspecifics in the same cage or enclosure.

For the following questions, please give the number of individuals of each species kept in each housing setting. (Since housing status can be fluid, please choose a single point in time and report the numbers for that particular day).

8. Singly housed (no physical contact but visual and olfactory contact provided)

Rhesus macaque

Long-tailed macaque

Pig-tailed macaque

Japanese macaque

Lion-tailed macaque

Stump-tailed macaque

Bonnet macaque

Baboons

Mangabeys

African green/vervet monkey

Patas monkey

Common squirrel monkey

Capuchins

Titi monkeys

Common marmoset

Cotton-top tamarin

Owl monkeys

Other species

9. Protected contact (separation of individuals via a barrier consisting of bars or mesh that permit social contact but not entry into the other individual’s cage)

Rhesus macaque

Long-tailed macaque

Pig-tailed macaque

Japanese macaque

Lion-tailed macaque

Stump-tailed macaque

Bonnet macaque

Baboons

Mangabeys

African green monkey

Patas monkey

Common squirrel monkey

Capuchins

Titi monkeys

Common marmoset

Cotton-top tamarin

Owl monkeys

Other species

10. Continuous pair housing

Rhesus macaque

Long-tailed macaque

Pig-tailed macaque

Japanese macaque

Lion-tailed macaque

Stump-tailed macaque

Bonnet macaque

Baboons

Mangabeys

African green monkey

Patas monkey

Common squirrel monkey

Capuchins

Titi monkeys

Common marmoset

Cotton-top tamarin

Owl monkeys

Other species

11. Intermittent pair housing (animals are paired regularly during part of the day/week)

Rhesus macaque

Long-tailed macaque

Pig-tailed macaque

Japanese macaque

Lion-tailed macaque

Stump-tailed macaque

Bonnet macaque

Baboons

Mangabeys

African green monkey

Patas monkey

Common squirrel monkey

Capuchins

Titi monkeys

Common marmoset

Cotton-top tamarin

Owl monkeys

Other species

12. Indoor group housing

Rhesus macaque

Long-tailed macaque

Pig-tailed macaque

Japanese macaque

Lion-tailed macaque

Stump-tailed macaque

Bonnet macaque

Baboons

Mangabeys

African green monkey

Patas monkey

Common squirrel monkey

Capuchins

Titi monkeys

Common marmoset

Cotton-top tamarin

Owl monkeys

Other species

13. Indoor group housing with access to the out of doors

Rhesus macaque

Long-tailed macaque

Pig-tailed macaque

Japanese macaque

Lion-tailed macaque

Stump-tailed macaque

Bonnet macaque

Baboons

Mangabeys

African green monkey

Patas monkey

Common squirrel monkey

Capuchins

Titi monkeys

Common marmoset

Cotton-top tamarin

Owl monkeys

Other species

14. Outdoor group housing

Rhesus macaque

Long-tailed macaque

Pig-tailed macaque

Japanese macaque

Lion-tailed macaque

Stump-tailed macaque

Bonnet macaque

Baboons

Mangabeys

African green monkey

Patas monkey

Common squirrel monkey

Capuchins

Titi monkeys

Common marmoset

Cotton-top tamarin

Owl monkeys

Other species

15. If some NHPs are singly housed, what reasons prevent them from being housed socially? Answer: Yes, No, Not applicable (i.e device is not used).

	Yes	No	N/A
Wearing of cranial implants (e.g. headposts, recording chambers, implanted arrays)	○	○	○
Wearing of internal implants in the body cavity	○	○	○
Wearing of eye coils	○	○	○
Other types of implants (e.g. vascular access ports)	○	○	○
Wearing of collars	○	○	○
Wearing of jackets	○	○	○
Use of tethering (e.g. for intravenous infusion)	○	○	○
Requirement for total urine collection	○	○	○
Requirement for measurement of individual food/fluid consumption	○	○	○
Monitoring of clinical signs (e.g. vomiting)	○	○	○
Use of restricted/controlled diets (food or fluid)	○	○	○
Concerns about cross-infection between animals (infectious disease or vaccine studies)	○	○	○
Anticipated rapid clinical decline	○	○	○
Other scientific justification (please specify below)	○	○	○
Time or staff constraints	○	○	○
Exemption on welfare grounds as advised by veterinarian/NACWO (e.g. when an animal is judged hyper-aggressive or considered excessively socially impaired)	○	○	○
Long-term clinical exemption	○	○	○
Concern about potential negative consequences to NHPs (e.g., injury, stress). Animals have not been formally exempted, behaviorally or clinically, or otherwise approved by Institutional Animal Care and Use Committee, Animal Welfare Body or Research Ethics Committee (hereafter IACUC/AWB/REC)	○	○	○
Cost	○	○	○
Housing or space limitations	○	○	○
Incompatibility of animals/unavailability of potentially compatible social partners	○	○	○
Information from research investigators is lacking (e.g., treatment groups, viral status)	○	○	○
Single housing required imminently	○	○	○
Belief that it does not benefit well-being	○	○	○

Other (please specify):

[ ]

16. Who is responsible for social management (including determining group membership, conducting social introductions, monitoring for initial and ongoing compatibility)? (tick all that apply)

	Introductions in cages	Introductions in outdoor enclosures
Behavioral management, veterinary, or research technician(s)	◯	◯
Behavioral management scientist(s)	◯	◯
Researcher(s)	◯	◯
Veterinarian(s)	◯	◯
Welfare Officer(s) / Facility Manager(s)	◯	◯

Other (please specify):

17. Is behavior recorded as part of social management?

- ◯ No
- ◯ Yes, qualitatively
- ◯ Yes, quantitatively
- ◯ Yes, both qualitatively and quantitatively

18. In pair introductions, is the observation of affiliative behavior a requirement for deeming an introduction successful?

- ◯ Yes
- ◯ No

19. Which of the following techniques do you ever employ when introducing NHPs? (tick all that apply)

- ☐ Use of compatibility/hierarchy data from the breeder/supplier
- ☐ Initial phase of visual access (e.g. through clear material or fine mesh) before permitting physical contact
- ☐ Protected contact phase prior to full contact
- ☐ Introduction of caged NHPs in neutral cages (i.e., cages that are new to all animals)
- ☐ Introduction of large groups in neutral enclosures
- ☐ Introduction in enclosures larger than primary housing
- ☐ Introduction in enclosures smaller than primary housing (to allow quick separation in case of aggression)
- ☐ Introduction of related animals where possible (e.g. siblings, half-siblings)
- ☐ Medication administered with the goal of improving outcome of the introduction (e.g. anesthesia)
- ☐ Group formations involving introductions of individuals or subgroups prior to the formation of the entire group
- ☐ Canine blunting
- ☐ Contraception
- ☐ Further comments on social housing:

Nursery rearing

Nursery rearing: purposeful separation of infants from their dams before 1 year of age and rearing them without the dam.

20. Do you employee nursery rearing at your facility?

- No
- Yes - with the aim of increasing animal production
- Yes - for the derivation of specific-pathogen free colonies
- Yes - for assignment to research projects requiring infants as part of the experimental model

21. If infants are removed from dams with the aim of increasing animal production, at what minimum age are infants removed?

0 months 12

22. If infants are removed for the derivation of specific pathogen-free colonies, at what minimum age are infants removed?

0 months 12

23. If infants are removed for assignment to research projects requiring infants as part of the experimental model, at what minimum age are infants removed?

0 months 12

24. Does your IACUC/AWB/REC ever approve single housing for infants?

- Yes
- No

25. Are nursery reared animals ever housed in single cages for any reason other than severe medical conditions?

- No
- Yes
- If yes, please explain why:

26. What nursery rearing techniques do you utilize? (tick all that apply)

- [ ] Single housing
- [ ] Peer rearing (continuous social housing with consistent group membership)
- [ ] Peer rearing (continuous social housing with rotating group membership)
- [ ] Surrogate / peer-rearing (continuous access to an inanimate surrogate with access to peers for part of the day)

27. Where single housing is used, what are the reasons for it? (tick all that apply)

- [ ] Approval from the IACUC/AWB/REC
- [ ] Severe medical conditions or maternal abuse/incompetence?
- [ ] Only for the first few days after birth to determine initial health condition
- [ ] Other (please specify):

28. At what age do singly-housed animals transition to continuous social housing?

0 months 12

29. For rearing in peer groups, what group size do you aim for?

30. Are socially-housed infants exposed to all social partners at the same time or is group/pair composition rotated among familiar partners?

- ( ) All partners at the same time
- ( ) Group/pair composition is rotated

Positive reinforcement training

Positive reinforcement training: teaching NHPs to voluntarily perform desired behaviors by providing rewards immediately following the performance of the behavior and with no use of coercion (e.g., cage squeeze mechanism or physical restraint).

31. What proportion of NHPs is provided at least some positive reinforcement training?

	NHPs in cages	NHPs in outdoor enclosures
None	○	○
1 to 50%	○	○
>50 to 99%	○	○
All	○	○

32. If applicable, what constrains the use of positive reinforcement training for all NHPs at your facility? (tick all that apply)

- [ ] Cost
- [ ] Personnel safety concerns
- [ ] Time or staff constraints
- [ ] Efficiency of training
- [ ] Research protocol exemption
- [ ] Concern about potential negative consequences to animals (e.g., injury, stress)
- [ ] Current training goals are pertinent only to a subset of animals
- [ ] Lack of expertise
- [ ] Belief that it will not be effective
- [ ] Belief that it does not benefit well-being
- [ ] Other (please specify):

33. Does your facility employ a dedicated training coordinator who is involved in positive reinforcement training across multiple research projects and the animal care department, and advises other individuals who train animals?

○ Yes

○ No

34. Who conducts positive reinforcement training at your facility? (tick all that apply)

- [ ] Dedicated trainer(s)
- [ ] Animal care, veterinary, or research technician(s)
- [ ] Behavioral management technician(s)
- [ ] Behavioral management scientist(s)
- [ ] Researcher(s)
- [ ] Veterinarian(s)
- [ ] Other (please specify):

35. Do personnel ever have direct physical contact with NHPs in the course of training, e.g. touching the body surface of the animal?

- ○ Yes
- ○ No

36. What positive reinforcement training goals are pursued? (tick all that apply)

- [ ] Cooperation with research procedures
- [ ] Cooperation with clinical procedures
- [ ] Cooperation with husbandry procedures, e.g., shifting animals to different enclosures
- [ ] Enhancing social housing, e.g., to reduce resource monopolization or improve social dynamics
- [ ] Reducing abnormal behaviors
- [ ] Reducing fearful or aggressive behavior in response to people or activities
- [ ] General enrichment (to provide stimulation, opportunities for problem solving, reduce boredom, etc.)
- [ ] Building trust and rapport between staff and animals
- [ ] Other (please specify):

37. Is positive reinforcement training ever required by the IACUC/AWB/REC for certain research procedures?

- ○ Yes
- ○ No
- ○ Further comments on positive reinforcement training:

38. Negative reinforcement: teaching NHPs to voluntary perform desired behaviors by removing something unpleasant or undesirable immediately following the performance of the behavior.

Is negative reinforcement ever employed in the course of attaining specific training goals? (Examples of negative reinforcement include using the squeeze-back cage mechanism or showing a net or hose during training sessions).

- ○ Yes
- ○ No
- ○ Further comments on negative reinforcement training:

Positive human interaction

Positive human interaction: engaging with NHPs in activities such as playing, grooming, other friendly interaction or handing out treats; excluding animal training and routine “rounds” to assess animal health.

39. What proportion of NHPs is provided with positive human interaction?

	NHPs in cages	NHPs in runs, field cages, or other enclosures
None	◯	◯
1% to 50%	◯	◯
>50% to 99%	◯	◯
All	◯	◯

40. If applicable, what constrains the use of positive human interaction for all NHPs? (tick all that apply)

- ☐ Cost
- ☐ Personnel safety concerns
- ☐ Time or staff constraints
- ☐ Research protocol exemption
- ☐ Concern about potential negative consequences to animals (e.g., injury, stress)
- ☐ Belief that it does not benefit well-being
- ☐ Other (please specify):

41. Who participates in this component of behavioral management?(tick all that apply)

- ☐ Animal care, veterinary, or research technician(s)
- ☐ Behavioral management technician(s)
- ☐ Behavioral management scientist(s)
- ☐ Researcher(s)
- ☐ Veterinarian(s)
- ☐ Other (please specify):

42. Do personnel ever have physical contact with NHPs in the course of human interaction?

- ○ Yes
- ○ No
- ○ What is the health status of the NHPs?

[ ]

Enclosure sizes

43. What proportion of your caged NHPs are provided more than the minimum applicable regulatory/accreditation-related floor space minimums?

- None
- 1% to 50%
- >50% to 99%
- All

44. Do you use European style pens at your facility?

- Yes - for all NHPs
- Yes - for some NHPs
- No

45. What proportion of indoor caged NHPs are provided access to exercise enclosures? (i.e. caging or pens that are not part of primary enclosure for animals housed at or near the regulatory/accreditation minimums; used to permit additional exercise or activity).

- None
- 1% to 50%
- >50% to 99%
- All

46. If exercise enclosures are not used for all indoor caged NHPs, what constrains its implementation? (tick all that apply)

- [ ] Cost
- [ ] Time or staff constraints
- [ ] Research protocol exemption
- [ ] Concern about potential negative consequences to animals (e.g., injury, stress)
- [ ] Space for the exercise enclosures
- [ ] Belief that it does not benefit well-being
- [ ] Other (please specify):

47. If exercise enclosures are used by some but not all indoor caged NHPs, what are the criteria for use? (tick all that apply)

- [ ] Singly housed animal in single cage
- [ ] Age (i.e. use is focused on immature animals)
- [ ] Intervention needed for undesirable behaviors
- [ ] Obesity
- [ ] Clinical needs/rehabilitation
- [ ] Compatibility with scientific procedure
- [ ] Other (please specify):

48. How often is a particular NHP typically given access to an exercise enclosure?

- ○ Daily
- ○ Less often than daily but several times a week
- ○ Once a week to once a month
- ○ Less than once per month
- ○ Other (please specify):

49. How long does a particular NHP typically remain in an exercise enclosure?

- ○ Up to 2 hours
- ○ >2 to less than 8 hours
- ○ >8 to less than 24 hours
- ○ >24 hours to one week
- ○ One to two weeks
- ○ Longer than two weeks
- ○ Other (please specify):

50. Further comments on exercise enclosures:

Destructible enrichment

Destructible enrichment: readily mutable material such as paper, cardboard, cloth, non-durable plastic, browse, straw.

51. What proportion of NHPs is provided with destructible enrichment?

	NHPs in cages	NHPs in runs, field cages, or other enclosures
None	○	○
1% to 50%	○	○
>50% to 99%	○	○
All	○	○

52. What proportion of NHPs is provided with floor substrate/bedding material?

	NHPs in cages	NHPs in runs, field cages, or other enclosures
None	○	○
1% to 50%	○	○
>50% to 99%	○	○
All	○	○

53. If applicable, what constrains the provision of destructible enrichment to all NHPs? (tick all that apply)

- ☐ Cost
- ☐ Plumbing or drainage concerns
- ☐ Other impact on sanitation
- ☐ Aesthetic concerns
- ☐ Time or staff constraints
- ☐ Research protocol exemption
- ☐ Concern about potential negative consequences to animals (e.g., injury, stress)
- ☐ Belief that it does not benefit well-being
- ☐ Other (please specify):

54. Further comments on destructible enrichment:

Sensory and cognitive enrichment

Sensory enrichment: passive enrichment features such as music, video, or aromas.
Cognitive enrichment: provision of challenging tasks intended to engage intelligence to solve problems (e.g. computer games, puzzle feeders).

55. What proportion of NHPs is provided sensory enrichment?

	NHPs in cages	NHPs in runs, field cages, or other enclosures
None	○	○
1% to 50%	○	○
>50% to 99%	○	○
All	○	○

56. If applicable, what constrains the provision of sensory enrichment to all NHPs? (tick all that apply)

- ☐ Cost
- ☐ Time or staff constraints
- ☐ Research protocol exemption
- ☐ Concern about potential negative consequences to animals (e.g., injury, stress)
- ☐ Belief that it does not benefit well-being
- ☐ Other (please specify):

57. Further comments on sensory enrichment:

58. What proportion of NHPs is provided cognitive enrichment?

	NHPs in cages	NHPs in runs, field cages, or other enclosures
None	○	○
1% to 50%	○	○
>50% to 99%	○	○
All	○	○

59. If applicable, what constrains the provision of cognitive enrichment to all NHPs? (tick all that apply)

- [ ] Cost
- [ ] Time or staff constraints
- [ ] Research protocol exemption
- [ ] Concern about potential negative consequences to animals (e.g., injury, stress)
- [ ] Belief that it does not benefit well-being
- [ ] Other (please specify):

60. Further comments on cognitive enrichment:

Behavioral pathology

Behavioral pathology: monitoring of and intervention for behavioral problems such as abnormal behavior, fearful behavior, or excessive aggression.

61. Are there individuals at your facility whose job it is to conduct regularly scheduled behavior observations (beyond daily health checks) to monitor for the presence of behavioral problems such as abnormal behavior, fearful behavior, excessive aggression? (tick all that apply)

- ☐ No, only as part of physical health checks
- ☐ Yes, conducted by animal care, veterinary, or research technician(s)
- ☐ Yes, conducted by behavioral management technician(s)
- ☐ Yes, conducted by behavioral scientist(s)
- ☐ Yes, conducted by researcher(s) or research staff
- ☐ Yes, conducted by veterinarian(s) during health checks
- ☐ Yes, other (please specify):

62. How often are scheduled behavior observations conducted (health checks excluded)?

	On caged NHPs	On NHPs housed in outdoor enclosures
Daily	○	○
Weekly	○	○
Monthly	○	○
Quarterly	○	○
Semi-annually	○	○
Annually	○	○
Not scheduled; only by referral	○	○

63. What kind of behavioral observations or assessments are conducted?

- ○ Qualitative observation noting presence or absence of any behavioral pathology
- ○ Quantitative behavioral observations (e.g., focal or scan sampling)
- ○ Other (please specify):

64. When behavioral pathologies are identified, are behavioral management techniques used as therapies to attempt to treat those pathologies?

- ○ Yes
- ○ No

65. If yes, who is responsible for deciding what techniques will be implemented for animals with behavior problem animals? (tick all that apply)

- [ ] Animal care, veterinary, or research technician(s)
- [ ] Veterinary technician(s)
- [ ] Behavioral management technician(s)
- [ ] Behavioral management scientist(s)
- [ ] Researcher(s)
- [ ] Veterinarian(s)
- [ ] Other (please specify):

66. Who is responsible for implementing therapeutic interventions for animals with behavior problems? (tick all that apply)

- [ ] Animal care, veterinary, or research technician(s)
- [ ] Behavioral management technician(s)
- [ ] Behavioral management scientist(s)
- [ ] Researcher(s)
- [ ] Veterinarian(s)
- [ ] Other (please specify):

67. What therapeutic interventions are employed? (tick all that apply)

- [ ] Re-location/re-grouping, PRT, additional enrichment, increased space, medication (e.g. anxiolytics / antidepressants), enhanced behavioural monitoring, etc
- [ ] Positive reinforcement training
- [ ] Additional environmental enrichment
- [ ] Increased enclosure space
- [ ] Medication (e.g. anxioloytics, antidepressants)
- [ ] Enhanced behavioral monitoring
- [ ] Other (please specify)

68. Following implementation of a therapeutic intervention, how is the success of the intervention assessed?

- ◯ No follow up occurs
- ◯ Weekly observation
- ◯ Monthly observation
- ◯ Quarterly observation
- ◯ Semi-annual observation
- ◯ Follow up frequency agreed with veterinarian/animal welfare officer
- ◯ Assessment of quantitative behavioral data
- ◯ Verbal reports from those working with the animal
- ◯ Other (please specify):

69. Further comments on intervention for behavioral pathology:

Employee training

Employee training: behavior and behavioral management training provided to staff.

70. Do new technicians receive behavioral management training by staff other than those who concentrate on the behavioral welfare programs? (tick all that apply)

- [ ] No
- [ ] Yes, by staff supervisor(s)
- [ ] Yes, by veterinary technician(s)
- [ ] Yes, by behavioral management technician(s)
- [ ] Yes, by behavioral scientist(s)
- [ ] Yes, by a research scientist(s) or staff
- [ ] Yes, by veterinarian(s)
- [ ] Yes, by other employee category (please specify):

71. If any components of behavioral management are specifically included in the job description of husbandry staff, what do they relate to? (tick all that apply)

- [ ] Social housing
- [ ] Positive reinforcement training
- [ ] Human interaction
- [ ] Structural enrichment
- [ ] Exercise enclosures
- [ ] Feeding enrichment
- [ ] Enrichment devices
- [ ] Other (please specify):
- [ ] Manipulable enrichment
- [ ] Destructible enrichment
- [ ] Sensory enrichment
- [ ] Cognitive enrichment
- [ ] Assessment of behavior
- [ ] Documentation of behavioral management activities
- [ ] Intervention for abnormal behavior

Program administration

Program administration: the behavioral management plan, program changes, and interface with IACUC/AWB/REC.

72. Do you have documentation of behavioral management strategies that are implemented? (tick all that apply)

- [ ] No
- [ ] Yes, a formal plan approved by the (IACUC/AWB/REC)
- [ ] Yes, Standard Operating Procedures containing information on behavioral management
- [ ] Yes, other internal documents (please specify):

73. In the last five years has anything prompted you to make changes to your behavioral management strategies? (tick all that apply)

- [ ] No
- [ ] Yes, an interested individual
- [ ] Yes, an internal review by the IACUC/AWB/REC
- [ ] Yes, suggestions or instructions from external site visitors (e.g., AAALAC, USDA, HO)
- [ ] Yes, a consultant
- [ ] Yes, other causes (please specify):
- [ ] Yes, changes in regulations or policies
- [ ] Yes, information in publications
- [ ] Yes, information from talking with colleagues
- [ ] Yes, information gained from conferences or workshops

74. Which aspects of behavioral management, if any... (tick all that apply)

	were changed in the past 5 years?	are you planning on enhancing in the next 6 months?
Social housing	○	○
Positive reinforcement training	○	○
Human interaction	○	○
Structural enrichment	○	○
Exercise enclosures	○	○
Feeding enrichment	○	○
Enrichment devices	○	○
Manipulable enrichment	○	○
Destructible enrichment	○	○
Sensory enrichment	○	○
Assessment of behavior	○	○
Documentation	○	○
Intervention for abnormal behavior	○	○
Employee training	○	○
Enrichment plan	○	○
Staffing levels and/or funding	○	○
IACUC/AWB/REC -related matters	○	○

Other (please specify):

75. If your IACUC/AWB/ERC addresses behavioral management issues, how are these discussions weighed in importance?

- [ ] Specific behavioral management questions must be addressed in every protocol review
- [ ] Behavioral management issues are routinely discussed but there is no requirement that it be addressed for every protocol
- [ ] Behavioral management issues are occasionally discussed but not addressed for every protocol
- [ ] Behavioral management issues are rarely discussed but not addressed for every protocol
- [ ] The IACUC/AWB/ERC does not address behavioral management issues

76. Do you have an individual with expertise in behavioral management or psychological wellbeing on the IACUC/AWB/REC?

- Yes
- No

77. If not, is input from individuals with behavioral management expertise solicited by the IACUC/AWB/REC via a different mechanism?

- Yes
- No
- Further comments on programmatic issues:
